# Targeting DNA repair pathways with B02 and Nocodazole small molecules to improve CRIS-PITCh mediated cassette integration in CHO-K1 cells

**DOI:** 10.1038/s41598-023-29863-8

**Published:** 2023-02-22

**Authors:** Behnaz Rahmani, Mohammad Hassan Kheirandish, Samaneh Ghanbari, Abbasali Raz, Fahimeh Shamsi, Fatemeh Davami

**Affiliations:** 1 Department of Medical Biotechnology, Faculty of Medicine, Semnan University of Medical Sciences, Semnan, Iran; 2 Department of Medical Biotechnology, Biotechnology Research Center, Pasteur Institute of Iran, Tehran, Iran

**Keywords:** Biotechnology, Gene delivery, Molecular engineering

## Abstract

CRISPR-mediated integration could be used to develop the recombinant CHO (rCHO) cells by knock-in into the hotspot loci. However, low HDR efficiency besides the complex donor design is the main barrier for achieving so. The recently introduced MMEJ-mediated CRISPR system (CRIS-PITCh) uses a donor with short homology arms, being linearized in the cells via two sgRNAs. In this paper, a new approach to improve CRIS-PITCh knock-in efficiency by employing small molecules was investigated. Two small molecules, B02, a Rad51 inhibitor, and Nocodazole, a G2/M cell cycle synchronizer, were used to target the S100A hotspot site using a bxb1 recombinase comprised landing pad in CHO-K1 cells. Following transfection, the CHO-K1 cells were treated with the optimum concentration of one or combination of small molecules, being determined by the cell viability or flow cytometric cell cycle assay. Stable cell lines were generated and the single-cell clones were achieved by the clonal selection procedure. The finding showed that B02 improved the PITCh-mediated integration approximately twofold. In the case of Nocodazole treatment, the improvement was even more significant, up to 2.4-fold. However, the combinatorial effects of both molecules were not substantial. Moreover, according to the copy number and out-out PCR analyses, 5 and 6 of 20 clonal cells exhibited mono-allelic integration in Nocodazole and B02 groups, respectively. The results of the present study as the first attempt to enhance the CHO platform generation by exploiting two small molecules in the CRIS-PITCh system could be used in future researches to establish rCHO clones.

## Introduction

Chinese hamster ovary (CHO) cell line is the most frequently applied host cell system to produce recombinant therapeutic proteins. Random integration as the conventional approach for developing the recombinant CHO (rCHO) cells is affected by several drawbacks, including expression instability and clonal variation mainly caused by the position effect^1,2^.

Targeted integration as an alternative strategy has attracted more comments recently by promoting transgene integration in a controlled manner and reducing the clone-to-clone heterogeneity^2,3^.

Among targeted integration approaches, CRISPR-Cas9 was recently introduced as a targetable nuclease tool to mediate site-specific integration in the mammalian genome^4,5^. This system introduces a double-strand break (DSB) into the genome by Cas9 endonuclease, which is directed to the target site through the guide RNA (gRNA)^6^. Once the break is generated, the DNA repair machinery accumulates at the damaged site to repair the break and maintain the genome integrity. The non-homologous end-joining (NHEJ) is a default repair pathway that effectively introduces random insertions and deletions (indels) at the cleavage site. NHEJ is frequently employed to create the loss of function mutations^7,8^. Alternatively, if the repair template is provided, the homology-directed repair (HDR) takes action to exert the desired editing into the target site. This system could be applied in CHO cell line development to target the therapeutic genes into specific genomic loci. However, despite the NHEJ pathway, the HDR is inefficient and only active during the late S/G2 stages of the cell cycle. Furthermore, the HDR-based donor template requires long homology arms, making the design complex and expensive^7,9,10^. Microhomology-mediated end-joining (MMEJ) as an alternative end-joining pathway uses microhomology arms (20–40 bp) and could be exploited in the targeted integration. Despite the HDR, it is active during the M, G1, and early S phases, and owing to its short arms, donor design is simple and cost-efficient. This alternative pathway mechanistically recruits different factors from HDR and NHEJ. MMEJ is also likely to occur in HDR and NHEJ defective cells^11–13^. As these pathways compete for DSB repair, it is clear if one pathway is incapacitated, other pathways can compensate for DSB repair^14^.

Recently, the CRIS-PITCh (Precise Integration into Target Chromosome) system has been developed to deploy the MMEJ pathway in CRISPR-mediated knock-in^15^. This system uses in vivo donor linearization to liberate the transgene with the help of sgRNA target sequences flanking the short homology arms^16^. CRIS-PITCh system has several advantages over HDR-mediate knock-in due to the need for short microhomology arms and targeting a wide range of cells^13,17^. However, CRIS-PITCh is confronted with low knock-in efficiency.

A growing body of research has benefited from interferer small molecules to stimulate the desired pathways or inhibit the competitor ones. In one study, research has shown that RS-1 (a Rad51 stimulator) as an HDR-promoting small molecule was able to increase CRISPR/Cas9 and TALEN-mediated knock-in efficiency in pluripotent stem cells^18^. In another attempt, SCR7 was employed to inhibit ligase IV and block the NHEJ pathway, to improve HDR-mediated editing by CRISPR/Cas9 in human cancer cells^19^.

Favoring one pathway over others could also be accomplished by a group of small molecules to synchronize the cell at the specific phases of the cell cycle. Some small molecules in this context were utilized for cell cycle synchronization: mimosine, aphidicolin, and thymidine for synchronizing in G2/M phases, Lovastatin to block cells at early G1 in primary human cells^20^, and Nocodazole, which causes arrest at G2/M phase in HEK293T, human U2OS and human pluripotent stem cells^20–22^. Furthermore, Nocodazole has been reported as an MMEJ activator due to its M phase arresting function in U2OS cells^21^. Inhibition of HDR or NHEJ pathways as the MMEJ competitor can force the cell to further bias repair towards MMEJ^14^.

The RAD51 molecule is a critical factor in the HDR pathway. RAD51 has a critical role in searching and invading homologous DNA sequences and the performance of homolog recombination^23^. It seems that inhibition of RAD51 can abolish HDR occurrence and stimulate alternative repair pathways, including NHEJ and MMEJ. The B02 small molecule is recently introduced as a potent RAD51 inhibitor^24,25^. However, the effect of small molecule treatment in CRIS-PITCh targeted integration into CHO cells has not yet been addressed. Furthermore, the accumulative effect of their simultaneous use was not studied.

In this study, with intending to improve the CRIS-PITCh knock-in efficiency, we evaluated the individual and combinatorial effects of B02 and Nocodazole small molecules to target an RMCE cassette into the downstream region of S100A locus of CHO-K1 cells which was recently established as a safe harbor site for high-level targeted expression^26^.

## Material and methods

### sgRNA design and donor construct for CRIS-PITCh

**Figure 1:**
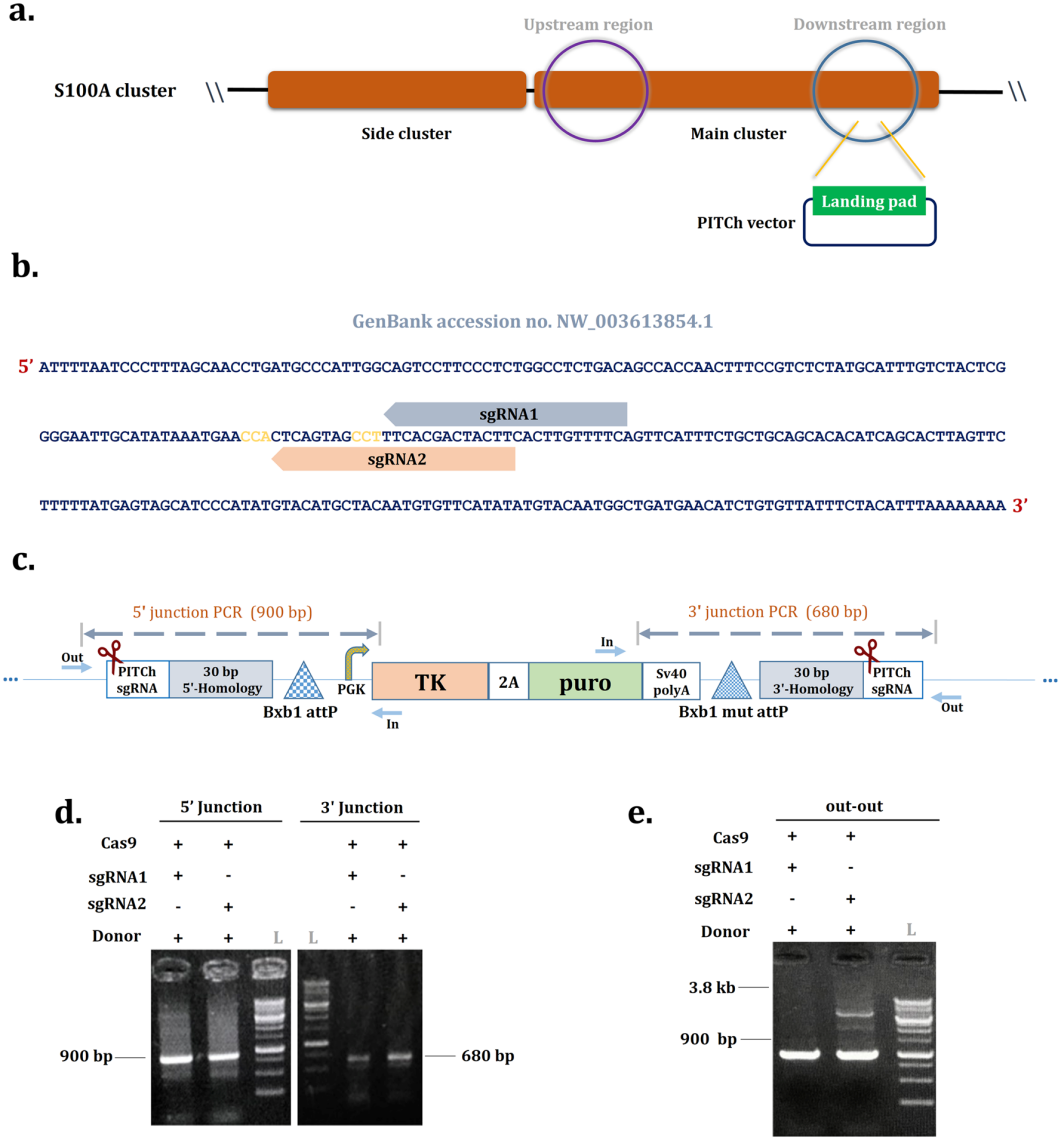
The PITCh-mediated integration of the RMCE landing pad into the S100A locus of CHO-K1. **(a)** The location of the S100A cluster. The downstream region of the main cluster was targeted by CRISP-PITCh. **(b)** The sgRNA1 and sgRNA2 target sites. The PAM sequence of each sgRNA was shown in yellow. **(c)** Schematic representation of CRIS-PITCh donor vector. The homology arms (30 bp) were located on both sides of the landing pad, and the PITCh sgRNAs flanked them. By Cas9-mediated cleavage of the donor, the MMEJ pathway proceeds with targeted integration. Primer position for junction and out-out PCRs is indicated. **(d)** Agarose gel of 5'/3' junction PCR results of stable cell pools of sgRNA1 and sgRNA2. The expected band size for 5' and 3' junction PCRs is 900 bp and 680 bp, respectively. L referred to the 1 kb DNA ladder. **(e)** Agarose gel of out-out PCR results of stable cell pools of sgRNA1 and sgRNA2. The locus-specific primers were used to amplify the whole integrated landing pad resulting in PCR product of the targeted locus band (3800 bp). The wild-type CHO band is 900 bp.

The CRIS-PITCh (v2) vector requires two sgRNAs for backbone-free cassette integration: (i) A single custom sgRNA targeting the intended genomic locus (downstream region of S100A cluster, NCBI accession number: NW_003613854.1), which is designed according to the CRISPOR sgRNA design online tool (http://crispor.tefor.net/) and (ii) A previously designed PITCh-gRNA^27^. The all-in-one backbone plasmid containing the Cas9 expression cassette and a gRNA scaffold was obtained from Addgene (Plasmid no. 64324). The construction procedures of the all-in-one vector (genomic sgRNA, PITCh sgRNA, and Cas9) are explained in a schematic illustration (Supplementary Fig. 1). Briefly, single-stranded oligos comprising the sgRNAs variable region were synthesized (Supplementary Table 1). They were annealed and mixed with the Bbs1 digested expression vector backbone, harboring U6 promoter and sgRNA scaffold sequence, to generate sgRNA expression vectors. For the CRIS-PITCh donor vector, the landing pad (attP-pGK-1-TK-2A-Puro-SV40pA-mut attP) was designed and ordered to be synthesized (Biobasic, Canada). The microhomology arms flanked the PITCh-gRNA target sequence. The size of the donor vector is 5743 bp (Fig. 1c).

### Cell culture and transfection

CHO-K1 cells (Cell Bank of Pasteur institute, Iran) were maintained in DMEM/F12 supplemented with 10% fetal bovine serum (Gibco, USA) and were incubated at 37˚C with 5% CO2. The cells were cultured in T flasks with a working volume of 5 ml and passaged every 3 days into fresh media. One day before transfection, CHO-K1 cells were seeded at a density of 6 × 10^4^ cells/well in 24-well plates containing 500 µl medium per well. The cells were co-transfected with donor plasmid (500 ng/µl) and all-in-one plasmid (500 ng/µl) using a lipofection 3000 reagent (Thermo Fisher, U.S.) according to the manufacturer’s protocol. The next day, transfected cells were checked with the fluorescent microscope (Zeiss Axioplan, Germany), then they were washed with PBS and fed by fresh medium.

### Small molecules compounds treatment

Commercially available small molecules used in this study were B02 (Cayman Chemical, CAY22133-5) and Nocodazole (SIGMA, m1404). Stock solutions of 25 mM for B02 and 1 mM for Nocodazole were made using dimethylsulfoxide (DMSO). The working solutions of each small molecule were prepared in the media with a final concentration of 0.05% DMSO.

### MTT assay

MTT assay was performed to evaluate the cytotoxicity of the B02 molecule on CHO-K1 cells. Briefly, the cells were plated in 96-well with DMEM F12 medium (~ 8000 cells/well) and incubated at 37 °C for overnight. Then, the cells were treated with B02 for 24 and 48 h at indicated concentrations. Next, the cells were washed 3 times with PBS solution, and then the MTT solution at the final concentration of 0.5 µg/ml was added to the respective wells and incubated for 4 h. The supernatant was discarded, and the formed formazan crystals were dissolved with 150 µl of 100% DMSO. The optical density (OD) of the wells was measured at 550 nm by spectrophotometer (Epoch, Biotek, USA).

### Cell cycle synchronization

Initially, the different conditions of treatment were analyzed in the CHO-K1 cell, in which the concentration of Nocodazole and treatment time were employed as the variables. The cells were treated with 40, 100, 200, 300, and 400 ng/ml of Nocodazole for 18, 24, and 30 h. Then the viability was considered; among them, just the concentration of 40 ng/ml did not significantly affect the cells’ viability (Fig. 3a).

Based on these results, more conditions were also analyzed to select the appropriate concentration and treatment time for cell cycle arrest. So, the 20, 40, 60, and 100 ng/ml of the small molecule were tested for 6, 12, 15, 20, 30, and 40 h to treat the cells. Once more, cell viability was analyzed (Supplementary Fig. 3), and accordingly, after Nocodazole removal, the cells with 40 and 60 ng/ml for 6, 12, and 15 h were in better condition than others. Therefore, a cell cycle arrest test was performed for these groups. According to the cell cycle synchronization results, the concentration of 40 ng/ml for 15 h was selected to follow (Fig. 3b). Based on *Gutschner’s* report^28^*,* 31 h after transfection, cells were treated with 40 ng/ml Nocodazole for 15 h, then washed 3 times with PBS, and transferred to 6-well plated with fresh media. One day later, the antibiotic selection was performed.

### Cell cycle analysis

15 h after Nocodazole treatment, cells were washed twice with PBS and suspended in 1 ml media. The cell cycle analysis was performed using flow cytometry assay (Cyflow) to estimate the percent of cells in each cell cycle phase.

### Generation and validation of stable cell line

48 h post-transfection, the cells were seeded at a density of 10^5^ cells/well into six-well tissue culture plates. After one day, the antibiotic selection was performed in a 2 ml medium containing 3 µg/µl of puromycin dihydrochloride (Biobasic, Canada) for 14 days. Following puromycin selection, the cell pools were harvested, and genomic DNA was extracted using a DNJia Plus Blood and Cell kit (Roje, Iran). To evaluate targeted cassette integration, 5'/3' junction PCR analysis was performed using PCR Master Mix (Ampliqon, Denmark) with specific primers to amplify cassette-locus boundary sites. The intact integration of a transgene was also confirmed with the out-out PCR using the super PCR master mix (YTA, Iran). The primers are listed in Supplementary Tables 2 and 3 and their positions are indicated in Fig. 1C.

The clonal selection was performed using the limiting dilution method. Briefly, the cells were trypsinized, collected, and adjusted to 5 cells/mL. Subsequently, 200 μL of the suspended cells were moved to each well of a 96-well plate to achieve 1 cell per well.

After limiting dilution, to verify the intact integration of an expression cassette into the targeted locus, PCR analysis was performed on the genomic DNA of each single-cell clone. To do so, the single-cell clones were expanded, harvested, and lysed according to the NaOH/Tris–HCl-based genomic DNA extraction protocol^29^: 1000 to 100,000 cells of each clone were centrifuged and cell pellets were resuspended in 20 μL of a 0.2 M NaOH solution. Then, the reaction was incubated at 75 °C for 10 min. Afterward, 180 μL of Tris–HCl solution with a concentration of 0.04 M (pH  7.8) was added per sample. The 2.5 μL of the prepared cell lysate was used as a template in 5′/3′ junction PCRs. 1% agarose gel was used to check the results. The 5′ or 3′ and 5′/3′ junction negative clones, were excluded from further examinations. The statistical significance was concluded with two-sided Fisher’s exact test on pairwise comparisons and data with P < 0.05 were considered statistically significant. The out-out PCR analysis was carried out on the kit-extracted genomic DNA of 5′/3′ junction positive clones using a pair of a primer specific to the outside of homology arms in the genomic locus.

### Copy number analysis, sequencing, and growth profile

The relative copy number of integrated thymidine kinase (TK) in single-cell clones was analyzed by quantitative real-time PCR (qRT-PCR) using the ABI 7500 System. The qRT-PCR was performed on genomic DNA samples in duplicate using RealQ Plus 2× Master Mix Green (Amplicon, Denmark). Amplification was executed with the following conditions: 95 °C for 10 min; 40× : 95 °C for 20 s, and 60 °C for 35 s to produce 135 bp amplicon from the TK gene. The primers were designed to encompass TK and beta-actin as the target and reference genes, respectively (Supplementary Table S4). All primers were validated empirically by melting curve analysis and agarose gel electrophoresis. Copy number was estimated using a delta–delta threshold cycle (ΔΔCt) method using a single-copy representative clone as the calibrator. The representative clone with a single copy of TK was determined by absolute real-time PCR as previously described^30^. Briefly, a standard curve with a tenfold serial dilution of linearized TK-comprised donor plasmid ranging from 10^8^ to 10^2^ copies/μL was plotted. The absolute copy number was estimated according to the equation:

$${\text{copy number of DNA }} = \frac{{{6}.0{22 } \times { 1}0^{{{23}}} \left( {{\text{copy}}/{\text{ mole}}} \right) \, \times {\text{ DNA amount }}\left( {{\text{ng}}} \right)}}{{{\text{DNA length }}\left( {{\text{bp}}} \right) \, \times { 66}0 \, \left( {{\text{Da}}} \right) \, \times { 1}0^{{9}} }}$$

To predict the TK copy number per CHO genome, the DNA content of each diploid cell was considered about 6 picograms owing to the reported 2.6 Gb CHO-K1 genome size^31^.

To asses any possible mutation in the targeted cassette, the 5′ and 3′ junction PCR amplicons of the nominated clones from each group were sent for Sanger sequencing using 5′ and 3′ junction reverse primers, respectively (sequencing primers are indicated in Supplementary Table 2).

For growth profile analysis, a single-copy clone from each group was selected, and its growth curve was plotted by determining the viable cell density for 8 days. To evaluate the effect of targeted knock-in on the growth profile, the doubling time and growth rate of each cell line were calculated during the exponential phase (between day 1, and day 4) as previously described^32^.

## Results

### The PITCh-mediated integration of the RMCE landing pad into the downstream region of the S100A locus (platform generation)

To check whether the CRIS-PITCh system could mediate cassette integration into the targeted locus, we exploited a CRIS-PITCh donor construct containing a landing pad flanked by the sgRNA target sequence (PITCh V2) and 30 bp microhomology arms adjoining both sides of the cassette. The landing pad was designed to develop a CHO cell platform. The cassette co-expresses the Herpes Simplex Virus-1 thymidine kinase (HSV-TK) and a puromycin (Puro) resistance gene driven by a PGK promoter. A pair of BXB1 attP sites were located on two sides of the cassette to perform BXB1 integrase-mediated RMCE (Supplementary Fig. 2). The S100A cluster, previously defined as an active transcriptional locus, served to create the CHO platform for sustained protein expression^26^.

At first, to select the sgRNA with an acceptable cleavage rate, two sgRNAs with the highest score in specificity and efficiency were selected according to the sgRNA online tools to target the downstream region of the S100A locus (Fig. 1a,b). Each sgRNA was cloned to the all-in-one vector and co-transfected to the CHO-K1 cells with the donor vector. Following puromycin antibiotic selection, the stable cell pools from each sgRNA were harvested, and 5'/3' junction PCR results showed on-target integration for both sgRNA groups (Fig. 1d). However, by performing out-out PCR with locus annealing primers to amplify the whole targeted cassette, only the sgRNA2 displayed the expected band size of 3800 bp (Fig. 1e). The wild-type CHO band (900 bp) in out-out PCR results could be as a result of either mono-allelic integration or random integration in a proportion of cells. Following the clonal selection of the sgRNA2 cell pool, a proportion of clonal cells were 5′/3′ junction positive (data not shown). Hence, the sgRNA2 (henceforth named sgRNA) was selected for further experiments.

### B02 and Nocodazole small molecule effects on PITCh-mediated knock-in efficiency

**Figure 2:**
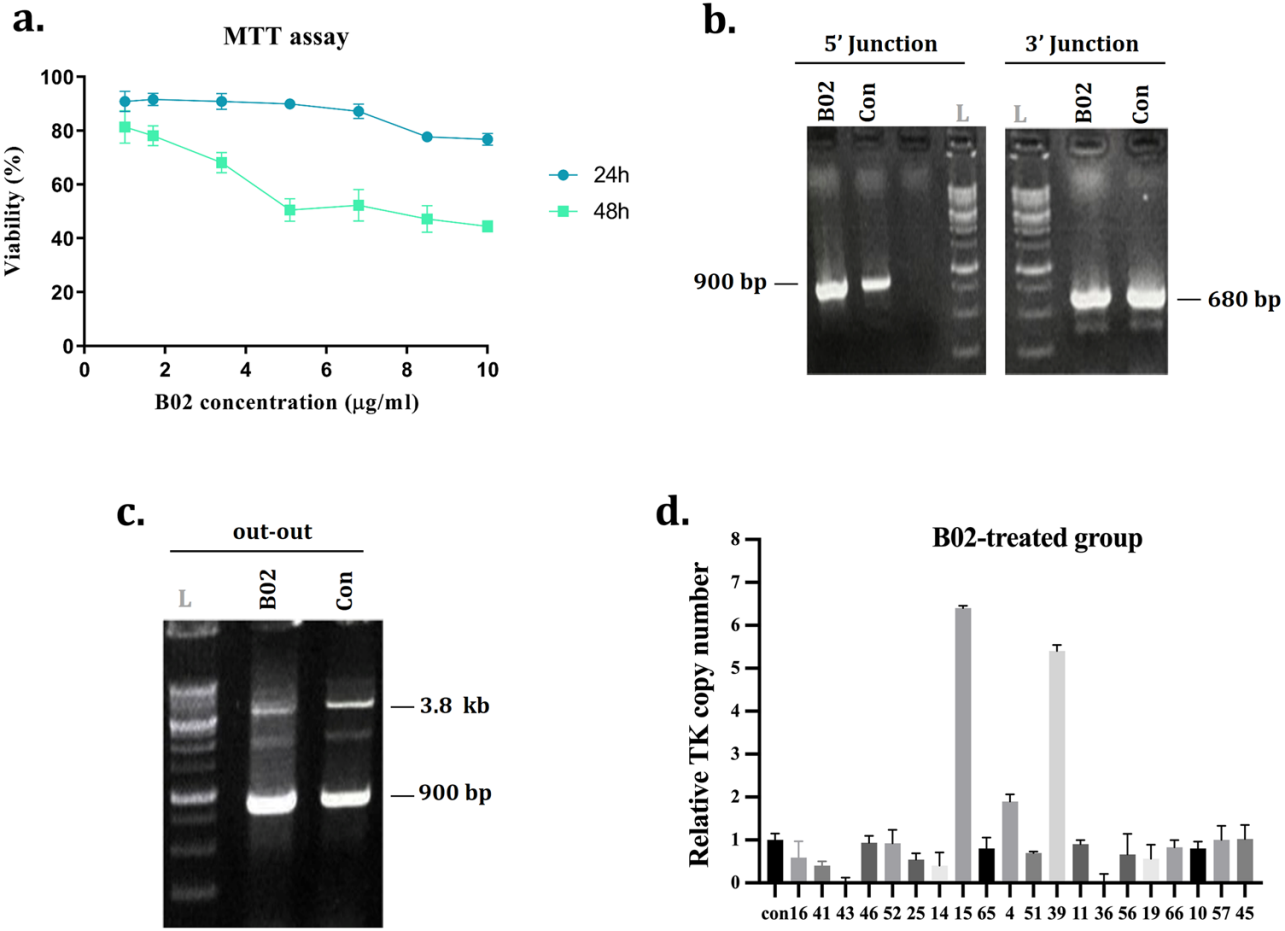
Evaluation of the B02 small molecule on the efficiency of PITCh-mediated integration. **(a)** The MTT viability assay was performed to evaluate the cytotoxicity of the B02 molecule on CHO-K1 cells. **(b)** Agarose gel of 5'/3' junction PCR results of stable cell pools of B02-treated and non-treated control groups. The expected band size for 5' and 3' junction PCRs is 900 bp and 680 bp, respectively. L referred to the 1 kb DNA ladder. **(c)** Agarose gel of out-out PCR results of stable cell pools of B02-treated and control groups. The expected PCR product band of the targeted locus and wild-type CHO is 3800 bp and 900 bp, respectively. **(d)** The relative copy number of thymidine kinase (TK) in B02-treated clonal cells. The error bars represent the standard deviations (n = 2).

To improve the CRIS-PITCh mediated knock-in efficiency, we exploited the B02 and Nocodazole small molecules. The B02 binds specifically to the Rad51 -a dominant molecule in the HDR pathway- and inhibits its function^24^. Since the MMEJ was known as the primary pathway for PITCh-mediated integration, we hypothesized that by inhibiting the HDR pathway using B02 small molecule, a higher knock-in efficiency might be achieved in this system. Firstly, to eliminate any potential toxicity of B02 on CHO-K1 cells, we evaluated the cytotoxic effects of different concentrations of B02 (1–8.5 µg/ml) for 24 and 48 h on the cells using an MTT assay (Fig. 2a). The MTT results showed the lowest viability drop at 1 µg/ml of B02. Besides, 48 h was selected since CRISPR operates during 24 to 48 h.

Cells were treated with 1 µg/ml B02 for 48 h, following the co-transfection with all-in-one and donor vectors. The cells were then washed with PBS and seeded in six-well plates. A group with the same condition except for B02 treatment was employed as the control. The stable cell pools were generated following puromycin selection and validated by 5′/3′ junction and out-out PCRs; as depicted in (Fig. 2b,c), each group showed a targeted knock-in within the S100A locus. By clonal selection procedure, 62 and 71 single-cell clones were recovered in the B02-treated and control groups, respectively; the genomic DNA of clonal cells was extracted and analyzed by 5′/3′ junction PCRs. The results determined that in the B02-treated group, 36 of 62 clones were both 5′ and 3′ junction PCR positive (58% KI efficiency). However, in the control group, 21 out of 71 clones were PCR positive (29% KI efficiency) (Supplementary Fig. 4a,b). Accordingly, B02 treatment resulted in an approximately twofold increase in CRIS-PITCh targeted integration efficiency (Table 1).

**Table 1:** The detailed results of knock-in efficiency in B02-, Nocodazole-, and double-treated groups.

Type of treatment	Total clone	Total PCRs	5′ junction PCR positive	3′ junction PCR positive	5'/3' junction PCR positive	KI efficiency (%)
Nocodazole-treated group	85	79	52	63	51	64
Control group	50	33	9	14	9	27
B02-treated group	70	62	37	55	36	58
Control group	74	71	22	55	21	29
Double-treated group	89	52	39	34	21	40
Control group	50	42	16	42	16	38

Subsequently, the Nocodazole was exploited to evaluate its effect on PITCh-mediated cassette integration owing to different action mechanisms compared to B02. This small molecule causes synchronization of the cells in the G2/M phase^21,22^. The HDR repair pathway is gradually terminated when the cells exit from the S phase, so it was assumed that G2/M arrested cells probably selected the MMEJ alternative pathway and caused an improvement in the PITCh-mediated knock-in efficiency.

**Figure 3:**
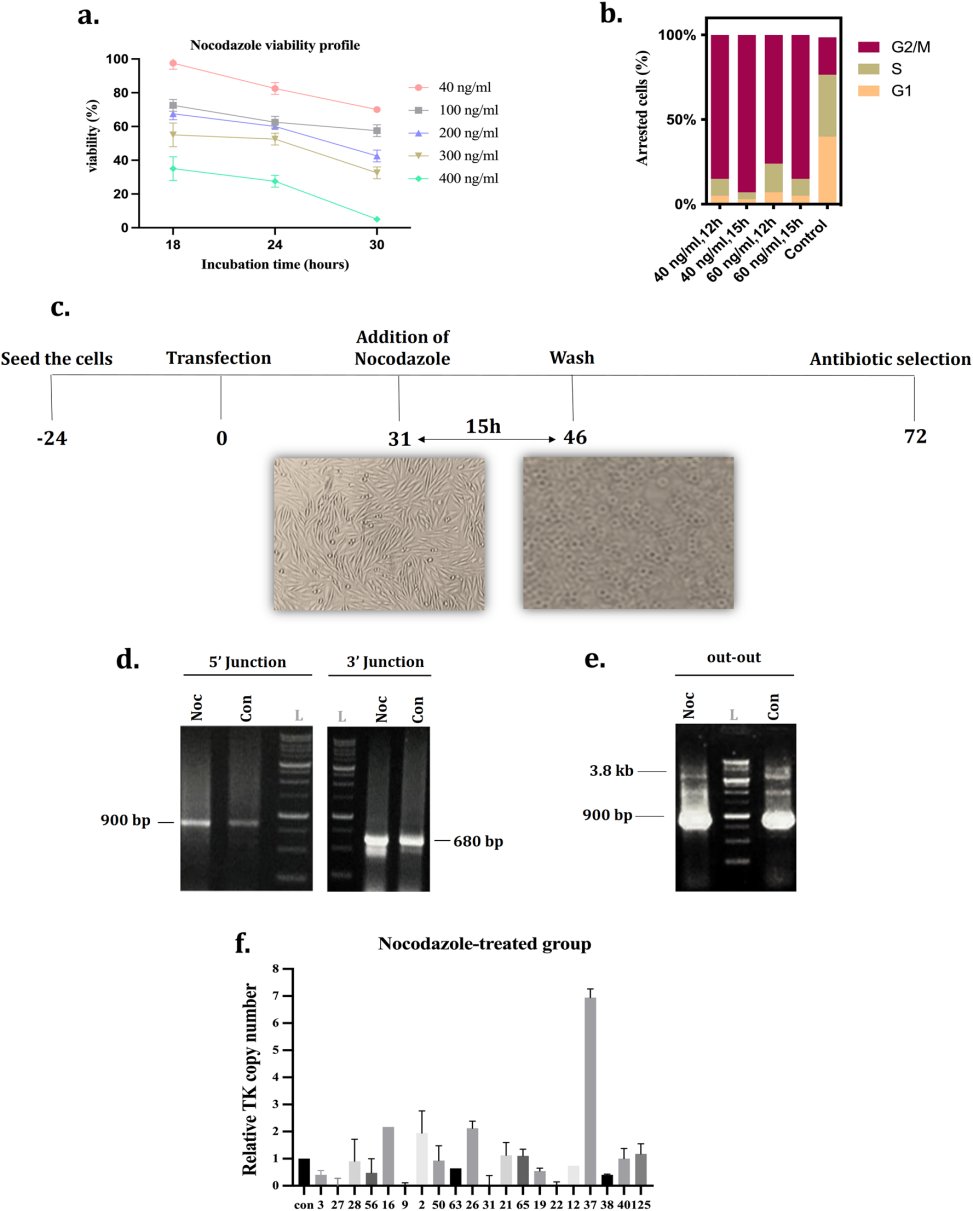
Evaluation of the Nocodazole small molecule on the efficiency of PITCh-mediated integration. **(a)** The cytotoxicity effect of 40–400 ng/ml Nocodazole on CHO-K1 cells during 30 h was determined by trypan blue staining assay. **(b)** The percent of arrested cells in each phase of the cell cycle following treatment with different concentrations of Nocodazole was shown by the stacked bar chart. The non-treated CHO-K1 was used as a control. **(c)** The timetable of Nocodazole treatment. The cells were seeded in 24-well, and 31 h post-transfection, they were treated with Nocodazole. The CHO-K1 cells morphologically changed from spindle-shaped to round cells during the treatment. **(d)** Agarose gel of 5′/3′ junction PCR results of stable cell pools of Nocodazole-treated and non-treated control groups. The expected band size for 5' and 3' junction PCRs is 900 bp and 680 bp, respectively. L: 1 kb DNA ladder, Noc: Nocodazole, Con: control. **(e)** Agarose gel of out-out PCR results of stable cell pools Nocodazole-treated and control groups. The expected PCR product band of the targeted locus and wild-type CHO is 3800 bp and 900 bp, respectively. **(f)** The relative copy number of thymidine kinase (TK) in Nocodazole-treated clonal cells. The error bars represent the standard deviations (n = 2).

40–400 ng/ml of Nocodazole which is routinely tested in different types of cells, was used to evaluate its toxicity effects on CHO cells (Fig. 3a). Based on the viability results, the cells were susceptible to the higher 100 ng/ml concentrations and treatment times above 24 h and were not recovered and showed abnormal morphologies. The concentration of 40 and 60 ng/ml were acceptable in terms of viability, so these concentrations were analyzed for cell cycle synchronization for 12 and 15 h of treatment by flow cytometry assay (Supplementary Fig. 5). The cells which were treated for 15 h at 40 ng/ml with Nocodazole exhibited 92.6% arresting in G2/M phases (Fig. 3b). Based on the synchronization assay results, 33 h after delivery of the all-in-one and donor vectors, cells were treated with 40 ng/ml Nocodazole for 15 h (Fig. 3c). Considering the transfection effect on cell cycle arrest, following transfection and Nocodazole treatment, Nocodazole-treated, and its non-treated control groups were checked regarding the cell cycle phase. Based on the flow cytometry results, about 88% of cells were arrested in G2/M phases in the presence of Nocodazole (40 ng/ml, 15 h) (Supplementary Fig. 5d). Cells were washed then and seeded in 6-well plates. After antibiotic selection, the cell pools of Nocodazole-treated and its control were collected and evaluated by PCR (Fig. 3d,e). Both pools revealed on-target intact cassette integration. Afterward, the clonal selection was carried out and 79 and 33 clones were recovered from Nocodazole-treated and its control group, respectively. According to the junction PCR results, 51 of 79 clones (64% KI efficiency) in the Nocodazole-treated group and 9 of 33 clones (27% KI efficiency) in the control group were 5′/3′ junction positive. Accordingly, these results suggest that Nocodazole treatment can increase knock-in efficiency up to 2.4-Fold (Table 1 and Supplementary Fig. 6a,b).

### Investigating the combinatorial effect of B02 and Nocodazole

To test whether the combination of B02 and Nocodazole enhances PITCh-targeted integration efficiency, we combined these small molecules and investigated their effect under the same condition. Following co-transfection of all-in-one and landing pad donor vectors, the cells were treated with 1 µg/ml B02 for 48 h. Additionally, 33 h post-transfection, they were treated with 40 ng/ml Nocodazole for 15 h. Indeed, the cells were exposed to both small molecules for 15 h. After washing with PBS, for small molecules removal, the cells were analyzed regarding the cell cycle phase, seeded in six-well plates, and the antibiotic selection was performed. The stable cell pool was verified by PCR analysis. After clonal selection, the targeted integration was analyzed on single-cell clones. Knock-in efficiency was calculated for the double-treated and control groups (without any Nocodazole or B02 treatment) (Supplementary Fig. 7). According to the 5'/3' junction result, the positive clones were measured to be 21 out of 52 clones in the double-treated group (40% KI efficiency) and 16 out of 42 (38% KI efficiency) in the control group. These results indicate that the combination of B02 and Nocodazole small molecules does not have a significant synergetic effect on the knock-in efficiency (Table 1 and Supplementary Fig. 8a,b).

### Investigating the effect of small molecules on random integration

The copy number of the TK transgene in clonal cells was estimated to investigate the effect of small molecules on random integration. The 20 single-cell clones with 5'/3' junction positive results were randomly selected from each group and assessed by qRT-PCR (Figs. 2d, 3f, and Supplementary Figs. 9 and 10). For better estimation, the results of real-time PCR were compared by out-out PCR results (Supplementary Figs. 11, 12 and 13). The being of a wild-type locus band (900 bp) with the targeted locus band (3.8 kb) could be interpreted as either mono-allelic or random integration. Eight clones in non-treated control (115, 20, 25, 32, 38, 19, 9, 18), five clones in Nocodazole (125, 38, 40, 65, 28), six clones in B02 (57, 52, 65, 56, 41, 19), and nine clones in double-treated (77, 56, 48, 14, 8, 42, 20, 71 and 86) groups demonstrated mono-allelic integration of landing pad cassette by presenting two bands in out-out PCR results and single-copy in quantitative PCR analysis. In contrast, clones with a single wild-type band in out-out PCR had different copy numbers and were concluded as the random integrants.

### Characterization of top clones by sequencing and growth profile analysis

**Figure 4:**
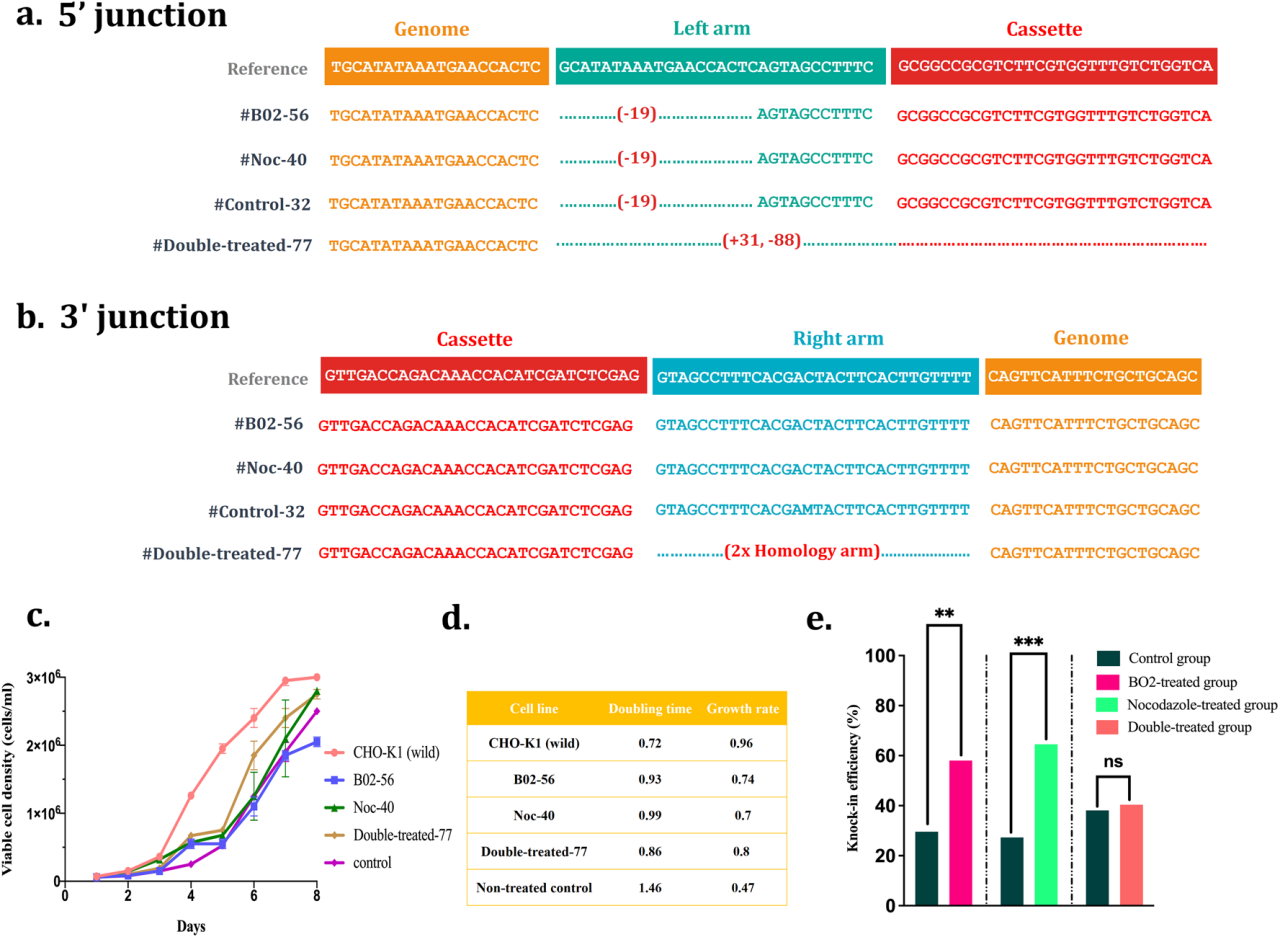
Sequencing results, growth profiles, and knock-in efficiency overview. **(a)** Single-copy clones from the Nocodazole group (Noc-40), B02 group (B02-56), double-treated group (Double-treated-77), and non-treated control group (Control-32) were selected randomly and their 5' junction PCR amplicons were sent for Sanger sequencing using 5' junction reverse primer. **(b)** The 3' junction PCR amplicons of selected clones were also sequenced using 3' junction reverse primer. **(c)** Cell growth profiles of selected clones during 8 days (n = 2). **(d)** The doubling time and growth rate of each cell line between day 1 and day 4. **(e)** Overview of knock-in efficiency results in B02-, Nocodazole- and double-treated groups. Double asterisk indicates significance (P < 0.01), triple asterisk indicates significance (P < 0.001) and ns indicates non-significance (P > 0.05).

4 single-copy clones were nominated for sequencing of 5'/3' junction regions; Clone 40 from the Nocodazole group, clone 56 from the B02 group, clone 32 from the control group, and clone 77 from the double-treated group. The sequencing results (Fig. 4a,b) highlighted that except for clone 77 of the double-treated group, all clones despite the deletions in their left homology arm, showed no indel mutations within the targeted cassette.

Moreover, these selected clones were investigated regarding growth profile compared to a wild-type CHO-K1 cell line. As illustrated in Fig. 4c,d, the integration of an exogenous DNA resulted in decreased growth rate, remarkably in the non-treated group.

## Discussion

The CRIS-PITCh is known as the MMEJ-interceded integration system to target predefined genomic sites via extremely short homology arms (20–40 bp) which can be easily prepared by PCR or synthetic oligonucleotides^33,34^. Here, in the initial step, we established a CHO cell line platform by targeting a nearly 2.6 kb RMCE landing pad cassette containing a pair of BXB1 attP sites into the S100A locus of the CHO-K1 genome through the CRIS-PITCh system. The CRIS-PITCh presents interesting superiorities such as straightforward design over conventional HDR-based approaches. However, since MMEJ is not a predominant DSB repair pathway in most of the cells^35^, for enhanced PITCh-mediated integration an active MMEJ pathway is required. Seeking to accelerate cell line development using CRIS-PITCh, we assumed that synchronizing the cells in MMEJ-permissive phases by a mediator molecule might improve the PITCh-mediated knock-in efficiency. As proposed by Hailong et al*.* arresting the cells in mitotic phases (G2/M) by the Nocodazole small molecule significantly increases the MMEJ activity while decreasing the HDR function^21^. Thus, we selected Nocodazole as a potential candidate to evaluate its effect on the CRIS-PITCh system for the first time. Consistently, in our experiment, the Nocodazole synchronized most of the CHO-K1 cells in MMEJ-permissive phases and consequently led to 64% PITCh-mediated knock-in efficiency. Almost 2.4-fold improvement was achieved compared to the non-treated control.

As a different approach, we investigated whether inhibition of the HDR -as an MMEJ competitor pathway- could enhance the DSB repair by MMEJ. To this end, we picked B02 small molecule with the previously-established HDR inhibitory effect via binding firmly to the RAD51 molecule^24^. The RAD51 accomplishes the essential step in the HDR pathway by creating DNA strand exchange. As hypothesized, the B02 small molecule showed nearly a twofold higher rate of PITCh-mediated knock-in compared to the non-treated control group (58% vs. 28%). This finding suggests that MMEJ may compensate for the lack of HDR in B02-treated cells to repair the DNA lesions.

Taken together, the Nocodazole and B02 small molecules individually showed a remarkable improvement in targeted cassette integration mediating by CRIS-PITCh via different action mechanisms, and we could introduce B02 and Nocodazole as novel CRIS-PITCh enhancer’s small molecules (overviewed in Fig. 4e).

However, unexpectedly, our analysis did not reveal any synergic effect between the two molecules. Our data demonstrated that there is no favorable collaboration on the molecular pathways relevant to Nocodazole and B02. Although there is no proof of concept behind that, according to our analysis (Supplementary Fig. 14) and previous findings^24,36,37^, the B02 molecule -unlike Nocodazole- arrests the cells in the G1 phase. Thus, we assumed that the lack of improvement in the knock-in rate might be as a result of signaling interference between them.

The evidence from this study suggests that small molecules with an approach to overcome the MMEJ pathway can be beneficial in competing with HDR to enhance the knock-in rate of the PITCh system. This is in agreement with Tomomi et al.’s research, in which the overexpression of Exo nuclease 1 as an MMEJ-enhancer increases PITCh-mediated knock-in efficiency in human cells by 2.5-fold^33^.

Copy number analysis together with out-out PCR results revealed some considerable results. For example, while clones 11 of the B02 group and 37 of the Nocodazole group were out-out PCR negative, they showed more than one copy number in real-time PCR results, indicating the random integration. Besides that, the clones 4 of the B02 group and 26 of the Nocodazole group were mono-allelic based on the out-out PCRs, because of both wild type locus band (900 bp) and targeted locus band (3800 bp), however, the quantitative PCR results revealed the copy numbers of 1.9 and 2.12 for them, respectively. Therefore, by inserting one copy in the target site and one copy in an off-target site, they were concluded as random integrants. The mono-allelic clones can be considered as the platform cell line for cell line development approaches.

The genotyping study of the 5′/3′ junctions, showed no mutations within the targeted cassette for all clones except for the double-treated group. The 3′ junctions of these clones also showed precise MMEJ-mediated integration, while the 5′ junctions were integrated by erroneous NHEJ.

The possibility of NHEJ-mediated integration is common in all linearized vectors^38^. Although in the case of intergenic integration or cell line development approaches like the present study, mutations within the adjacent sequences of cassettes are ineffective on the recombinant expression, it seems that of the inhibiting NHEJ pathway might enhance precise 5′/3′ junctions.

According to the growth profile analysis, all targeted clones showed higher doubling time and lower growth rates in comparison with a wild-type CHO-K1 cell line. This can be assumed as a result of the enhancing metabolic load caused by a heterologous expression of puromycin resistance and TK genes.

In summary, we showed that Nocodazole and B02 small molecules could promote rCHO cell line development by the CRIS-PITCh system with minimal toxicity. Generally, the overexpression of proteins involved in the MMEJ repair pathway or employment of small molecules to improve this pathway should be applied more in the future on CRIS PITCh as a feasible and highly efficient technology at the forefront of researchers’ use.

## Supplementary Information

Supplementary Information.

## Data Availability

All data generated or analyzed during this study are included in this published article.

## References

[1] Ronda C (2014). Accelerating genome editing in CHO cells using CRISPR Cas9 and CRISPy, a web-based target finding tool. Biotechnol. Bioeng..

[2] Lee JS, Kallehauge TB, Pedersen LE, Kildegaard HF (2015). Site-specific integration in CHO cells mediated by CRISPR/Cas9 and homology-directed DNA repair pathway. Sci. Rep..

[3] Bachu R, Bergareche I, Chasin LA (2015). CRISPR-Cas targeted plasmid integration into mammalian cells via non-homologous end joining. Biotechnol. Bioeng..

[4] Jang H-K, Song B, Hwang G-H, Bae S (2020). Current trends in gene recovery mediated by the CRISPR-Cas system. Exp. Mol. Med..

[5] Li H (2020). Applications of genome editing technology in the targeted therapy of human diseases: Mechanisms, advances and prospects. Signal Transduct. Target. Ther..

[6] Jiang F, Doudna JA (2017). CRISPR–Cas9 structures and mechanisms. Annu. Rev. Biophys..

[7] Yu W (2020). Repair of G1 induced DNA double-strand breaks in S-G2/M by alternative NHEJ. Nat. Commun..

[8] Lieber MR (2010). The mechanism of double-strand DNA break repair by the nonhomologous DNA end-joining pathway. Annu. Rev. Biochem..

[9] Sakuma, T. *BMC Proceedings.* 1–2 (BioMed Central).

[10] Martínez-Gálvez, G., Manduca, A. & Ekker, S. C. MMEJ-based precision gene editing for applications in gene therapy and functional genomics. *bioRxiv* (2020). 10.1093/nar/gkaa1156 PMC7797032 33305328

[11] Yanik M (2018). Development of a reporter system to explore MMEJ in the context of replacing large genomic fragments. Mol. Ther. Nucleic Acids.

[12] Wang H, Xu X (2017). Microhomology-mediated end joining: New players join the team. Cell Biosci..

[13] Van Vu, T. (2021). CRISPR/Cas-based precision genome editing via microhomology-mediated end joining. Plant Biotechnol. J..

[14] Helleday T, Petermann E, Lundin C, Hodgson B, Sharma RA (2008). DNA repair pathways as targets for cancer therapy. Nat. Rev. Cancer.

[15] Nakade S (2014). Microhomology-mediated end-joining-dependent integration of donor DNA in cells and animals using TALENs and CRISPR/Cas9. Nat. Commun..

[16] Wierson WA (2020). Efficient targeted integration directed by short homology in zebrafish and mammalian cells. Elife.

[17] Sakuma T, Nakade S, Sakane Y, Suzuki K-IT, Yamamoto T (2016). MMEJ-assisted gene knock-in using TALENs and CRISPR-Cas9 with the PITCh systems. Nat. Protoc..

[18] Song J (2016). RS-1 enhances CRISPR/Cas9-and TALEN-mediated knock-in efficiency. Nat. Commun..

[19] Hu Z (2018). Ligase IV inhibitor SCR7 enhances gene editing directed by CRISPR–Cas9 and ssODN in human cancer cells. Cell Biosci..

[20] Lin S, Staahl BT, Alla RK, Doudna JA (2014). Enhanced homology-directed human genome engineering by controlled timing of CRISPR/Cas9 delivery. Elife.

[21] Wang H (2018). PLK1 targets CtIP to promote microhomology-mediated end joining. Nucleic Acids Res..

[22] Yiangou L (2019). Method to synchronize cell cycle of human pluripotent stem cells without affecting their fundamental characteristics. Stem Cell Rep..

[23] Liu M (2019). Methodologies for improving HDR efficiency. Front. Genet..

[24] Shkundina IS, Gall AA, Dick A, Cocklin S, Mazin AV (2021). New RAD51 inhibitors to target homologous recombination in human cells. Genes.

[25] Riesenberg S, Maricic T (2018). Targeting repair pathways with small molecules increases precise genome editing in pluripotent stem cells. Nat. Commun..

[26] Mueller, M.J., Jochen, Koenitzer, J., & Bernloehr, C. *Integration Sites in CHO Cells*. (2019).

[27] Kawabe Y (2018). Targeted knock-in of an scFv-Fc antibody gene into the hprt locus of Chinese hamster ovary cells using CRISPR/Cas9 and CRIS-PITCh systems. J. Biosci. Bioeng..

[28] Gutschner T, Haemmerle M, Genovese G, Draetta GF, Chin L (2016). Post-translational regulation of Cas9 during G1 enhances homology-directed repair. Cell Rep..

[29] Freire, C. *Genome Editing via CRISPR/Cas9 Targeted Integration in CHO Cells*. (2017).

[30] Lee C, Kim J, Shin SG, Hwang S (2006). Absolute and relative QPCR quantification of plasmid copy number in *Escherichia coli*. J. Biotechnol..

[31] Xu X (2011). The genomic sequence of the Chinese hamster ovary (CHO)-K1 cell line. Nat. Biotechnol..

[32] Shang M (2021). Investigating the influence of physiologically relevant hydrostatic pressure on CHO cell batch culture. Sci. Rep..

[33] Aida T (2016). Gene cassette knock-in in mammalian cells and zygotes by enhanced MMEJ. BMC Genomics.

[34] Iwao, R., Kawabe, Y., Murakami, M., Ito, A. & Kamihira, M. *MATEC Web of Conferences.* 07001 (EDP Sciences).

[35] Sfeir A, Symington LS (2015). Microhomology-mediated end joining: A back-up survival mechanism or dedicated pathway?. Trends Biochem. Sci..

[36] Nascakova Z (2021). Rad51 inhibition induces r-loop formation in early g1 phase of the cell cycle. Int. J. Mol. Sci..

[37] Alagpulinsa DA, Ayyadevara S, Shmookler Reis RJ (2014). A small-molecule inhibitor of RAD51 reduces homologous recombination and sensitizes multiple myeloma cells to doxorubicin. Front. Oncol..

[38] Sawatsubashi S, Joko Y, Fukumoto S, Matsumoto T, Sugano SS (2018). Development of versatile non-homologous end joining-based knock-in module for genome editing. Sci. Rep..

