# Supplementary material for: Targeting DNA repair pathways with B02 and Nocodazole small molecules to improve CRIS-PITCh mediated cassette integration in CHO-K1 cells
Source: Sci Rep. 2023 Feb 22;13:3116. doi: 10.1038/s41598-023-29863-8 (PMC9947112; doi:10.1038/s41598-023-29863-8)
Supplement: Supplementary file 1 — Supplementary Information. [file 41598_2023_29863_MOESM1_ESM.docx]

**
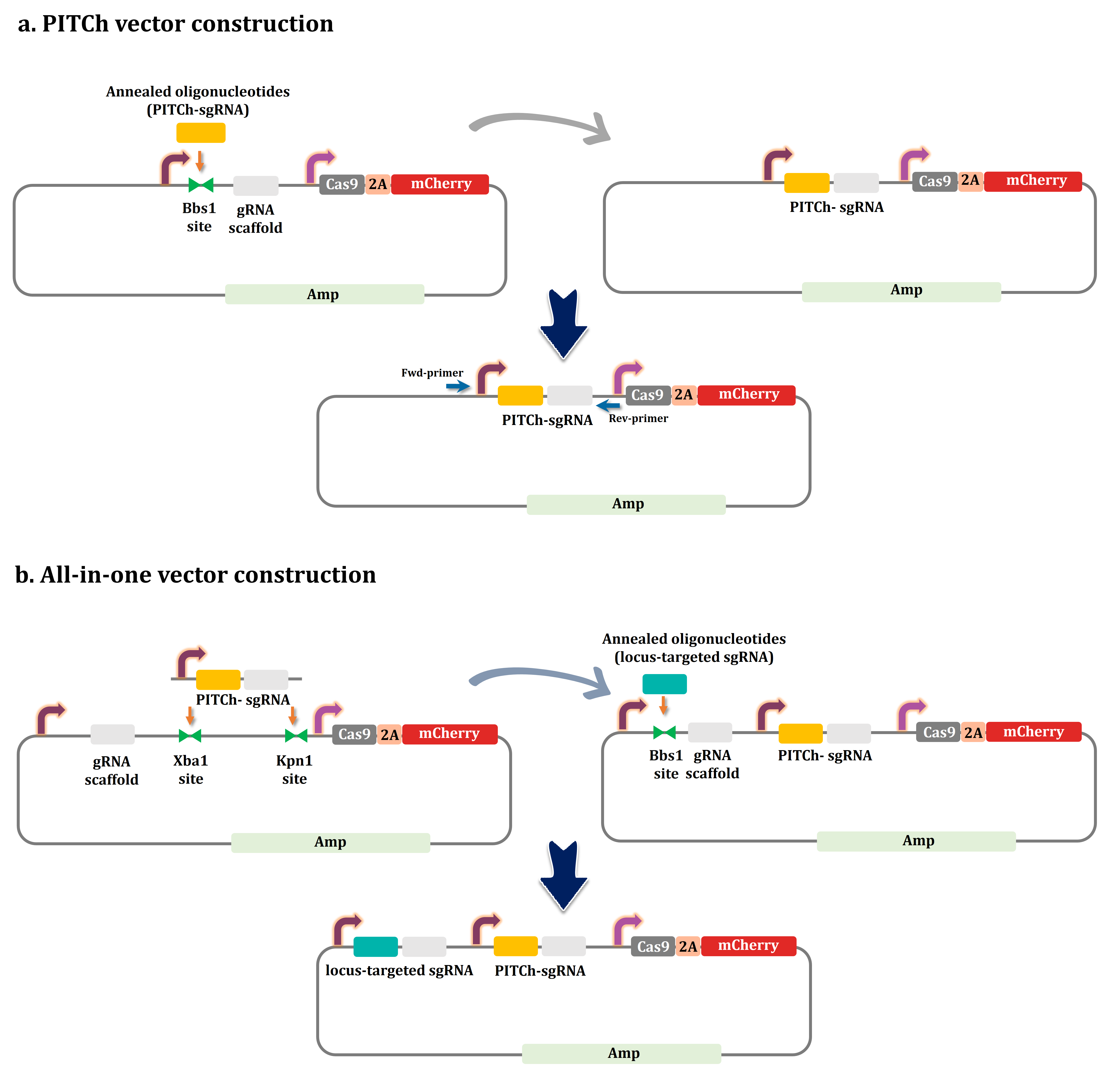
**

**Supplementary Figure 1. Schematic representation of all-in-one vector construction.** **(a)** The annealed PITCh sgRNA cloned to the vector comprising Cas9 expression cassette and sgRNA scaffold. **(b)** The PITCh sgRNA cassette was amplified by PCR, digested with Xba1 and Kpn1 restriction enzymes, and sub-cloned into the all-in-one vector double-digested with the same enzymes. Then the annealed locus-targeted sgRNA was cloned into the modified all-in-one vector.


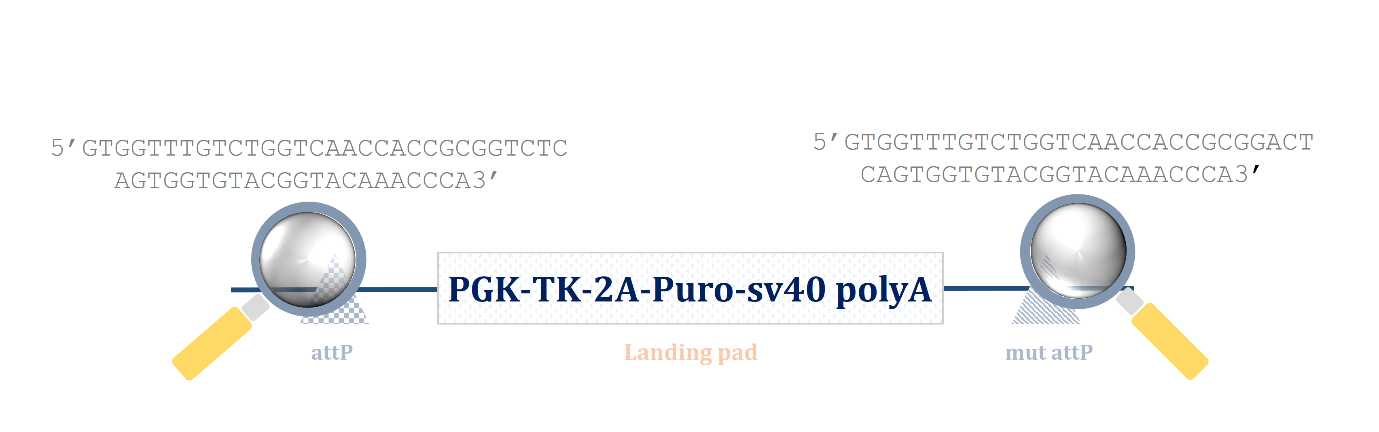


**Supplementary Figure 2. The sequence of BXB1 integrase attP sites.**

**
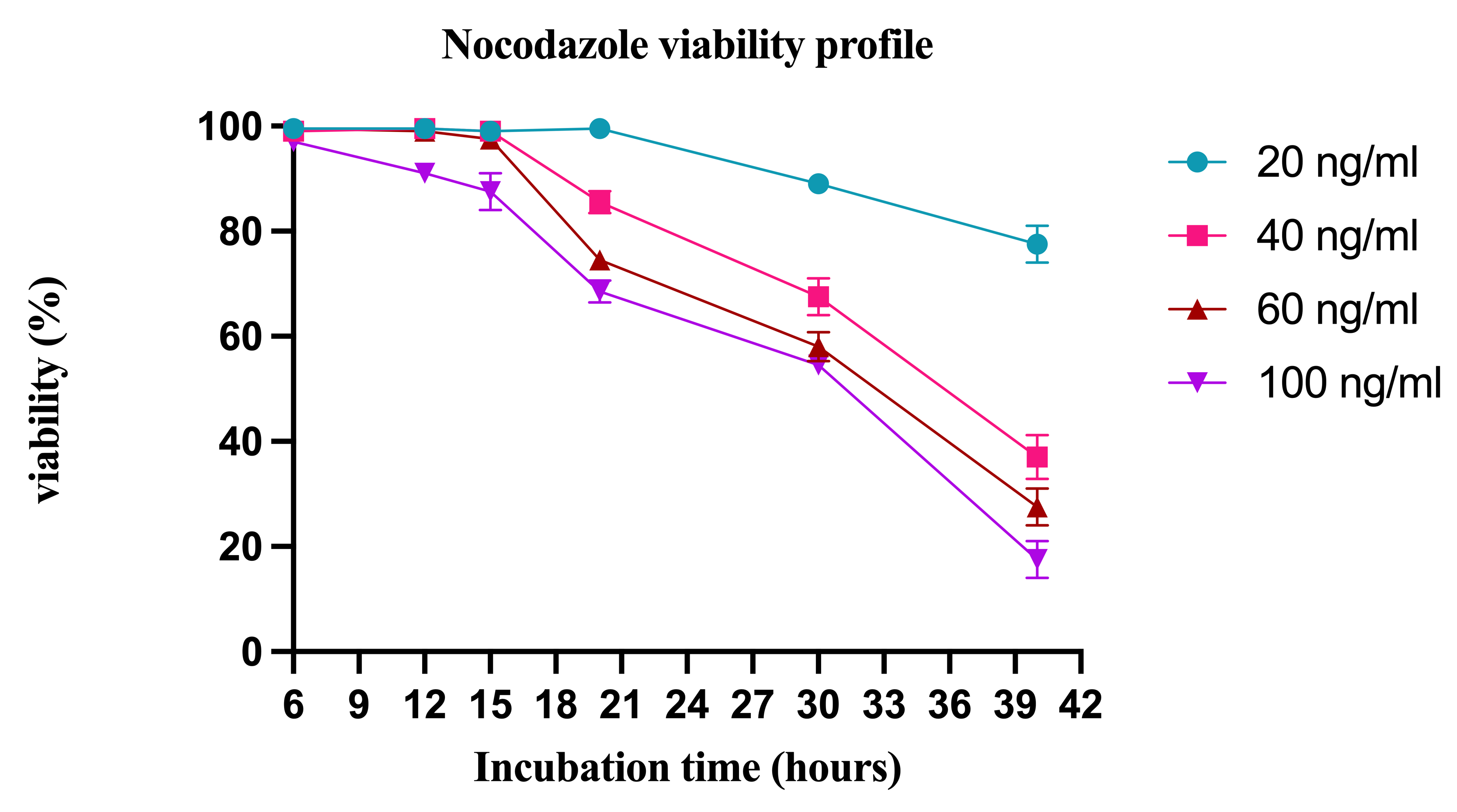
**

**Supplementary Figure 3. The cytotoxicity effect of 20-100 ng/ml Nocodazole on CHO-K1cells during 40 hours was determined by trypan blue staining assay.**

**
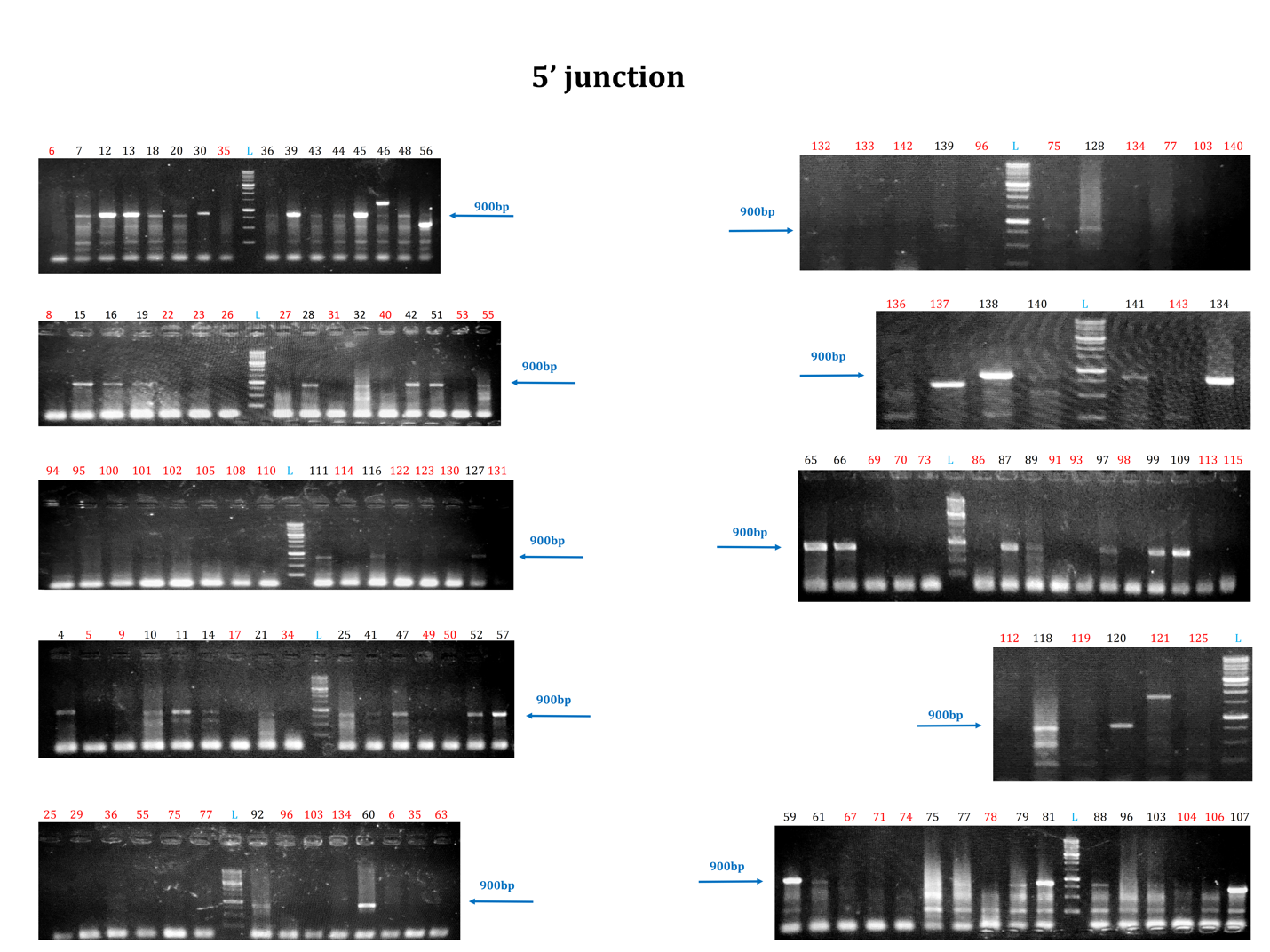
**

**Supplementary Figure 4a. Agarose gel electrophoresis of 5' junction PCR results of single-cell clones in the B02-treated group.** Clones 1 to 70 are B02-treated and 71 to 144 are non-treated control. The PCR or gel electrophoresis analysis of the faint band was repeated to catch the better band.

**
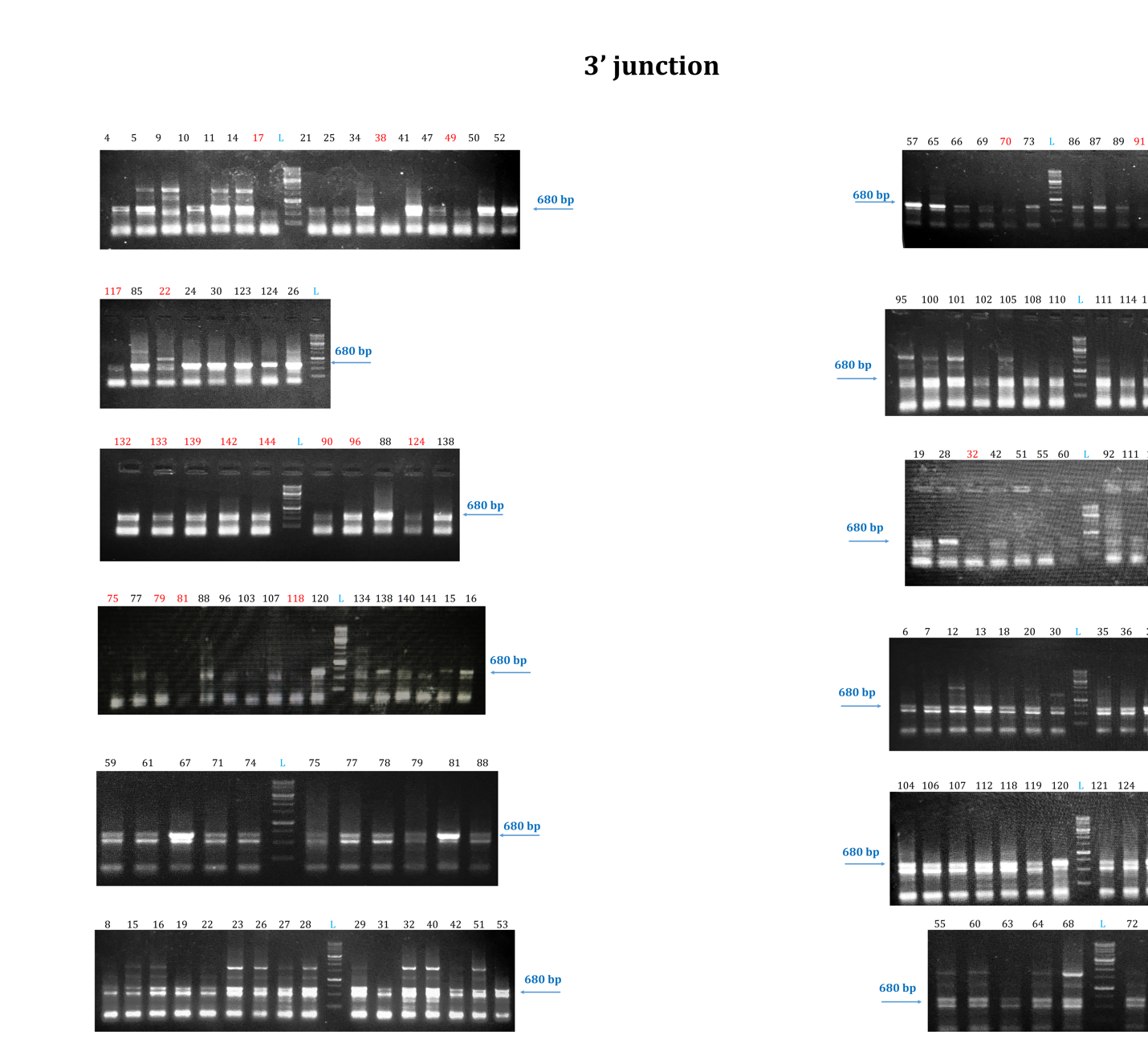
**

**Supplementary Figure 4b. Agarose gel electrophoresis of 3' junction PCR results on single-cell clones of the B02-treated group.** Clones 1 to 70 are B02-treated and 71 to 144 are non-treated control. The PCR or gel electrophoresis analysis of the faint band was repeated to catch the better band.

**
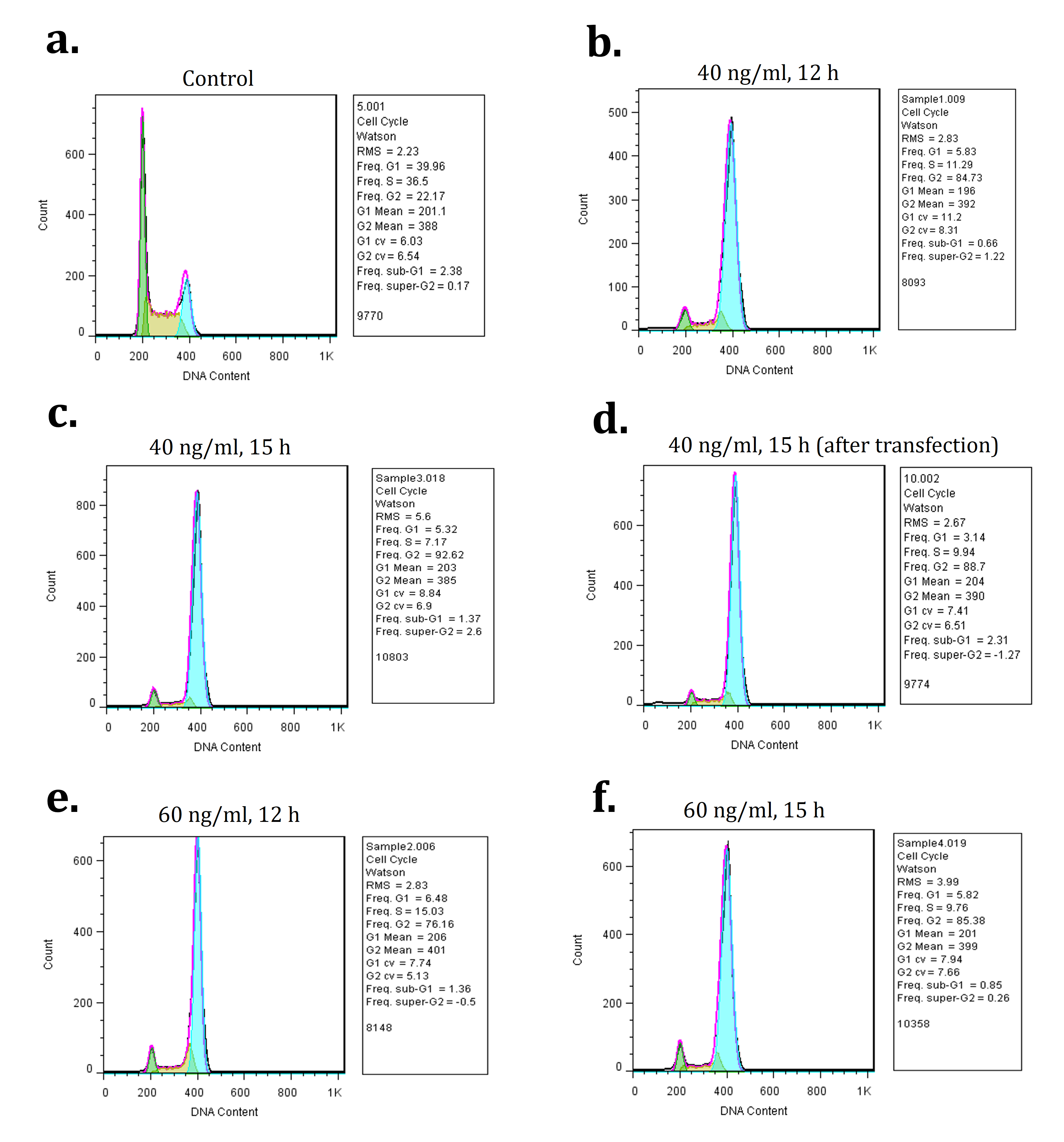
**

**Supplementary Figure 5. Cell cycle synchronization analysis following Nocodazole treatment by Flow cytometry assay. (a)** The CHO-K1 cell without any treatment was used as a control. **(b)** Treatment of cells with 40 ng/ml of Nocodazole for 12 hours. **(c)** Treatment of cells with 40 ng/ml of Nocodazole for 15 hours. **(d)** Treatment of cells with 40 ng/ml of Nocodazole for 15 hours following transfection. **(e)** Treatment of cells with 60 ng/ml of Nocodazole for 12 hours. **(f)** Treatment of cells with 60 ng/ml of Nocodazole for 15 hours.


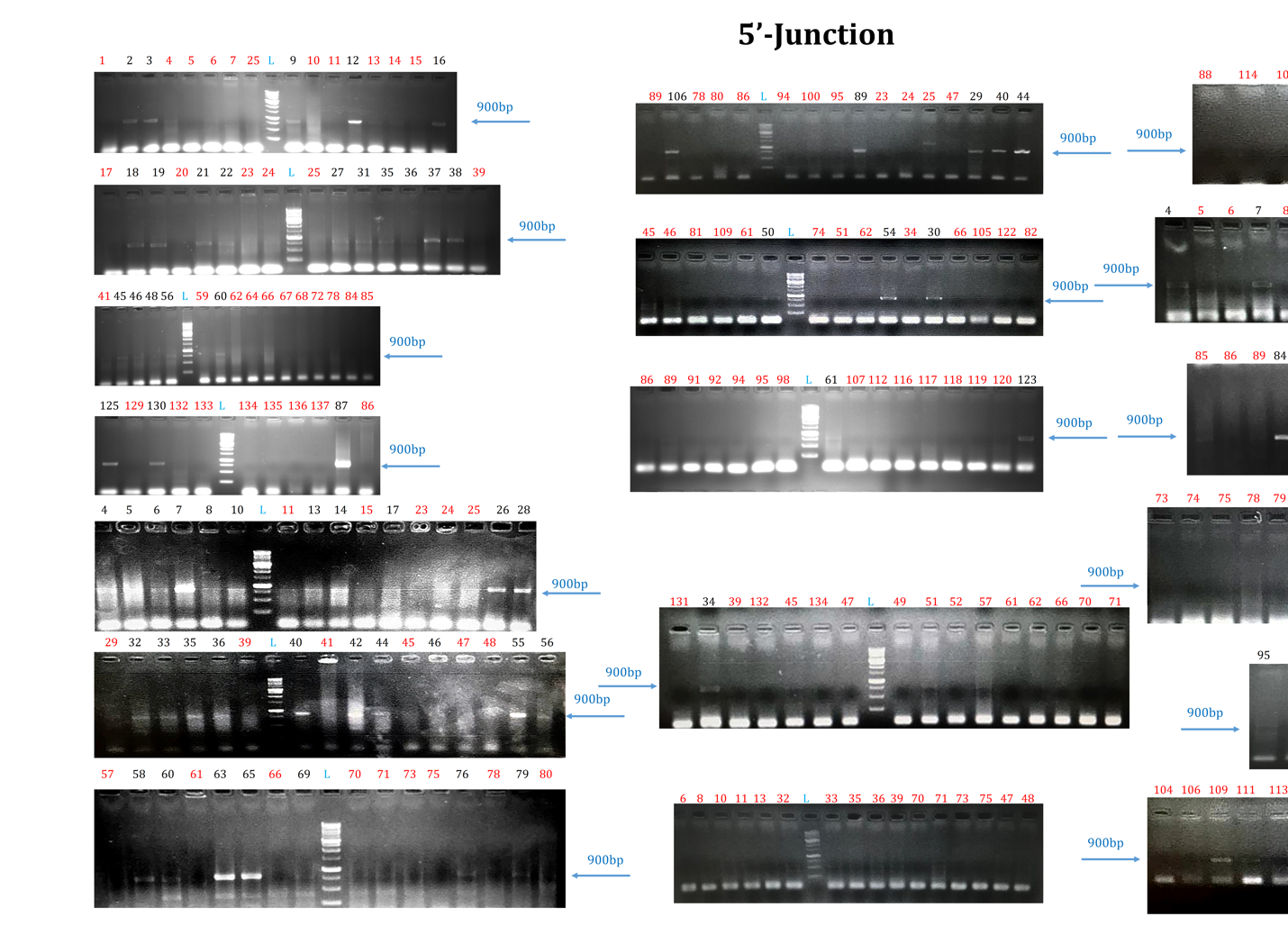


**Supplementary Figure 6a. Agarose gel electrophoresis of 5' junction PCR results on single-cell clones of the Nocodazole-treated group.** Clones 1 to 69 and 121 to 137 are Nocodazole-treated and 70 to 120 are non-treated control. The PCR or gel electrophoresis analysis of the faint band was repeated to catch the better band.

**
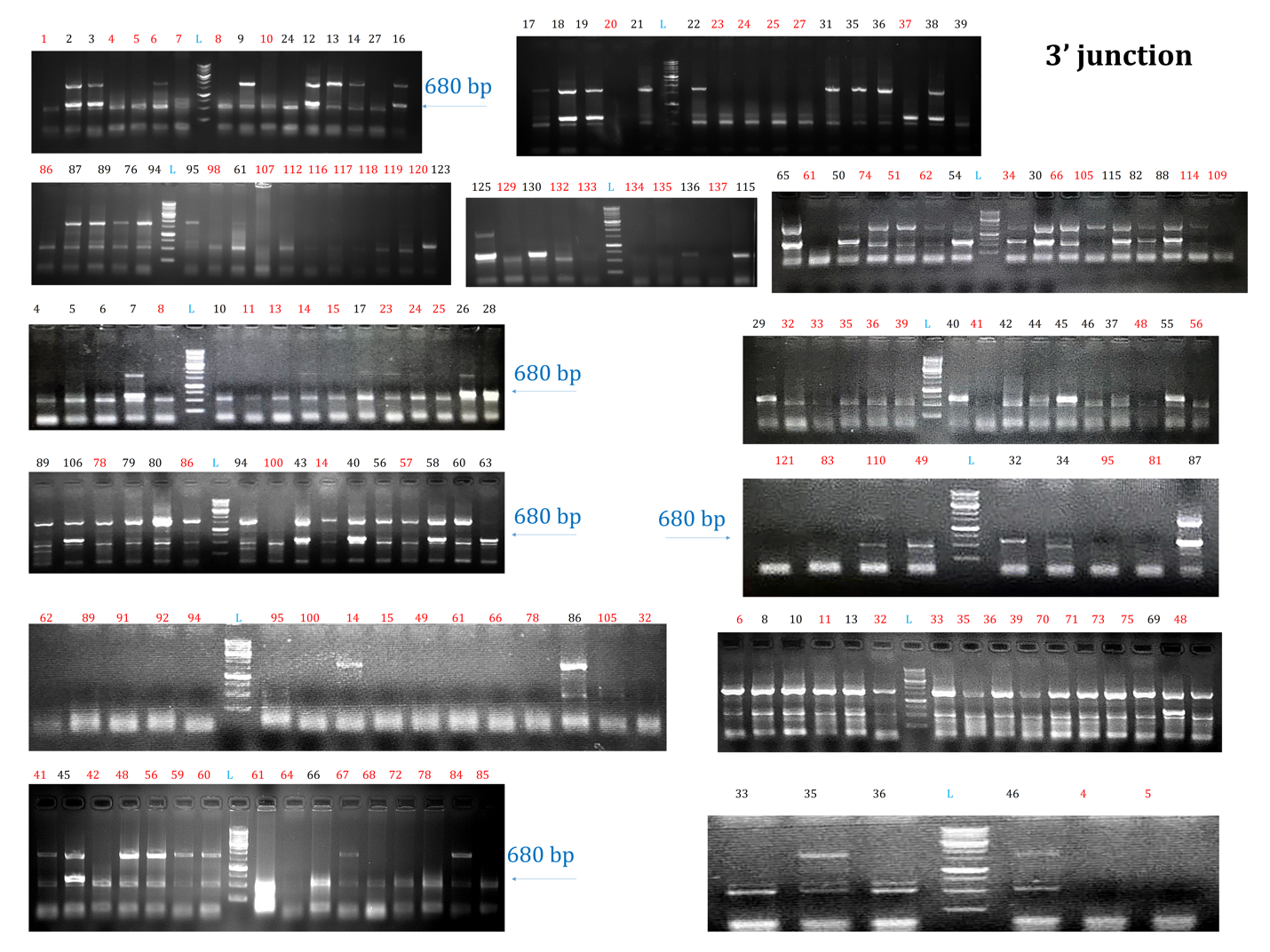
**

**Supplementary Figure 6b. Agarose gel electrophoresis of 3' junction PCR results on single-cell clones of the Nocodazole-treated group.** Clones 1 to 69 and 121 to 137 are Nocodazole-treated and 70 to 120 are non-treated control. The PCR or gel electrophoresis analysis of the faint band was repeated to catch the better band.

**
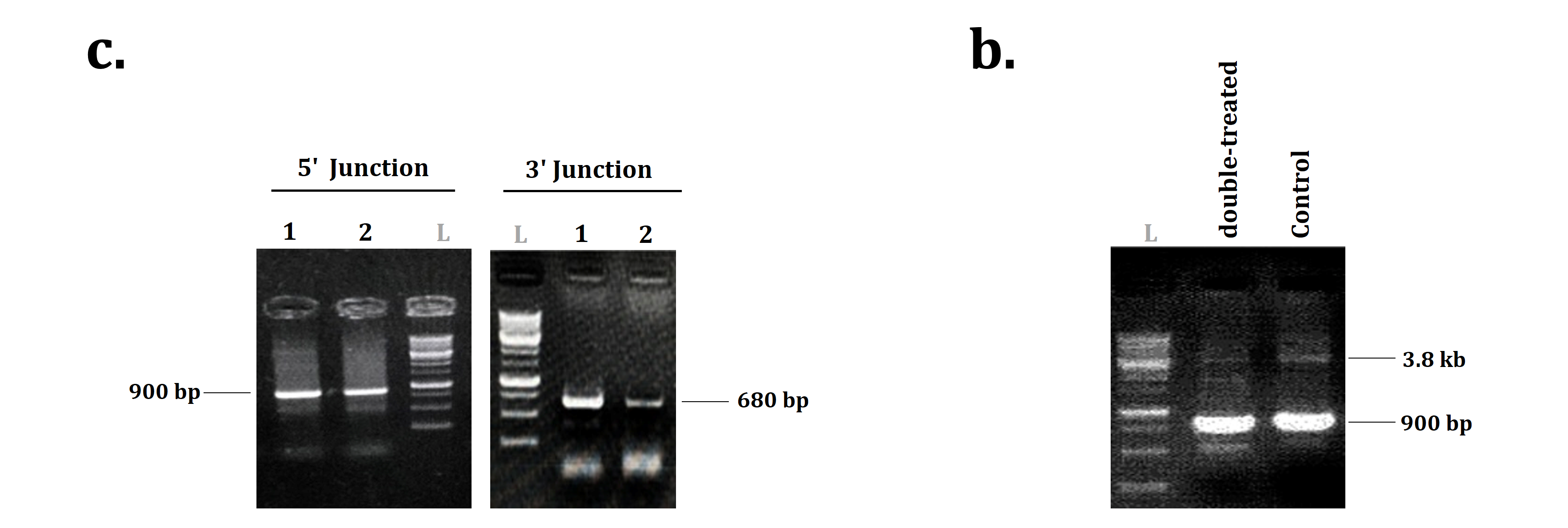
**

**Supplementary Figure 7. The results of 5'/3'** **junction and out-out PCRs of stable cell pools of double-treated** **group.** **(a)** Agarose gel of 5'/3' junction PCR results of stable cell pools of double-treated and non-treated control groups. Expected band size for 5' and 3' junction PCR is 900 bp and 680 bp, respectively. Lane 1, double-treated group; Lane 2, control group. L referred to 1 kb DNA ladder. **(b)** Agarose gel of out-out PCR results of stable cell pools of double-treated and control groups. The expected PCR product band of targeted locus and wild-type CHO is 3800 bp and 900 bp, respectively.


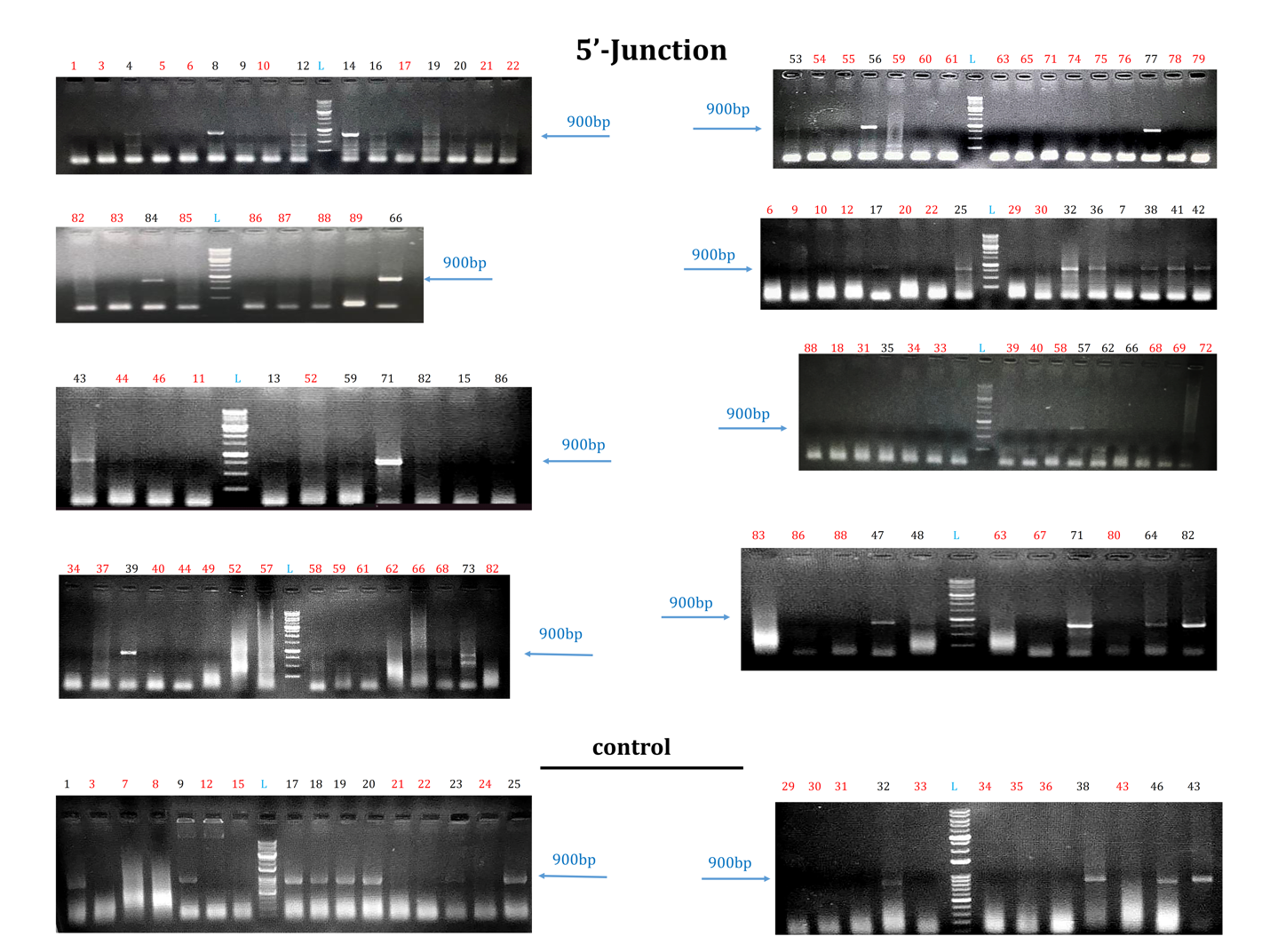


**Supplementary Figure 8a. Agarose gel electrophoresis of 5' junction PCR results on single-cell clones of the double-treated group.** Clones of double-treated (1-89) and non-treated control (1 to 50) were determined. The PCR or gel electrophoresis analysis of the faint band was repeated to catch the better band.

**
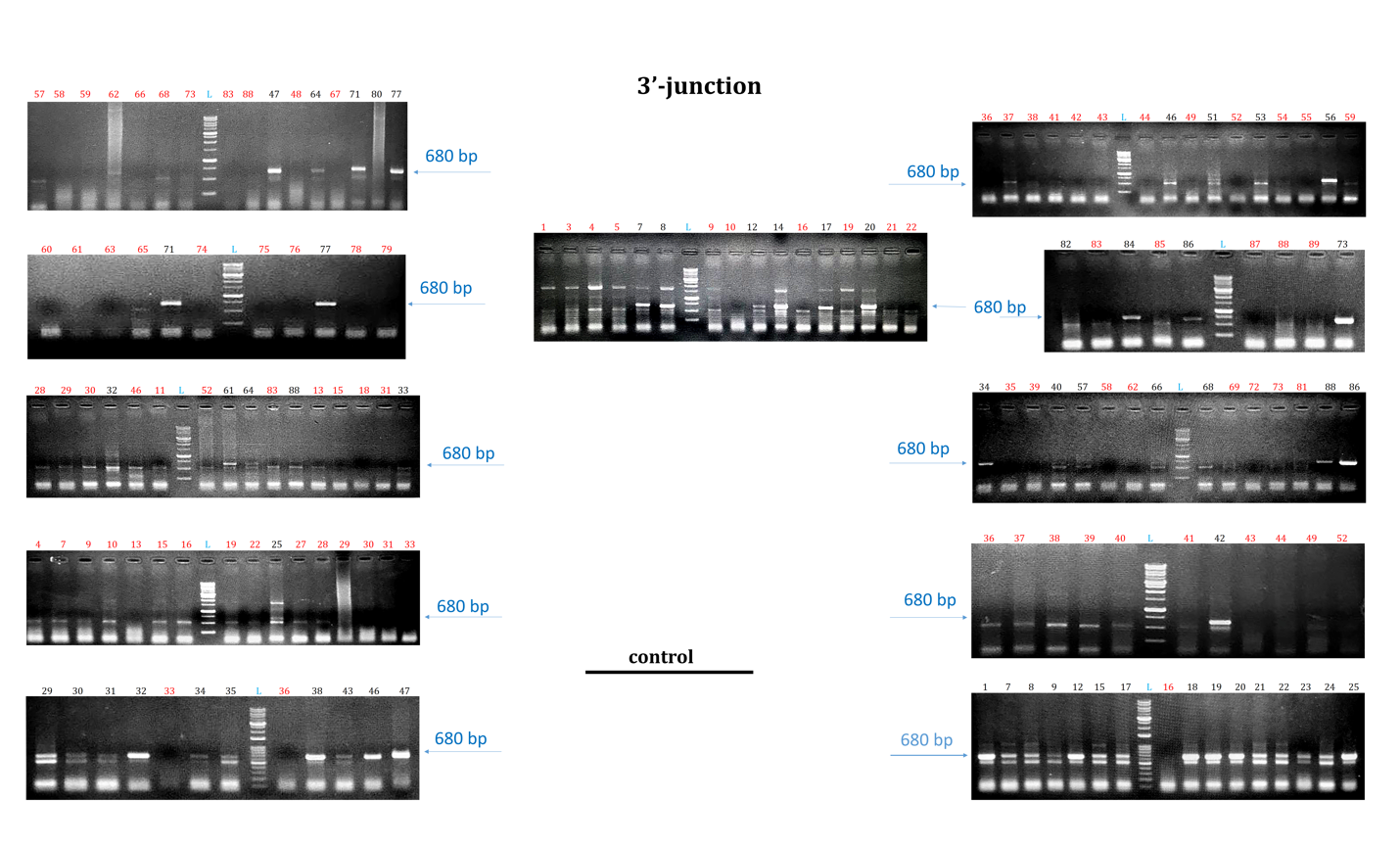
**

**Supplementary Figure 8b. Agarose gel electrophoresis of 3' junction PCR results on single-cell clones of double-treated group.** Clones of double-treated (1-89) and non-treated control (1 to 50) were determined. The PCR or gel electrophoresis analysis of the faint band was repeated to catch the better band.

**
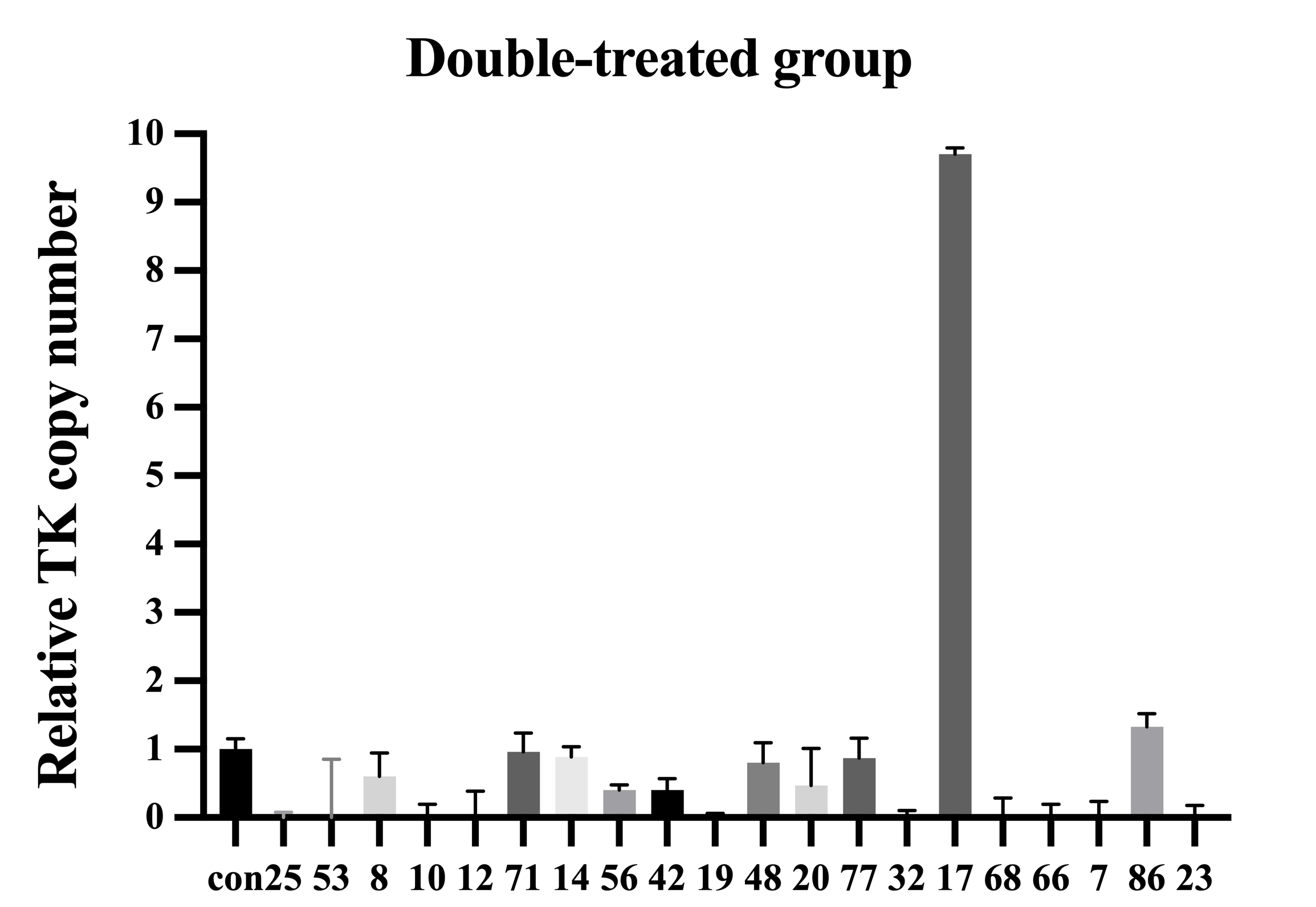
**

**Supplementary Figure 9. The relative copy number of thymidine kinase (TK) in double-treated group.** The error bars represent the standard deviations (n = 2).


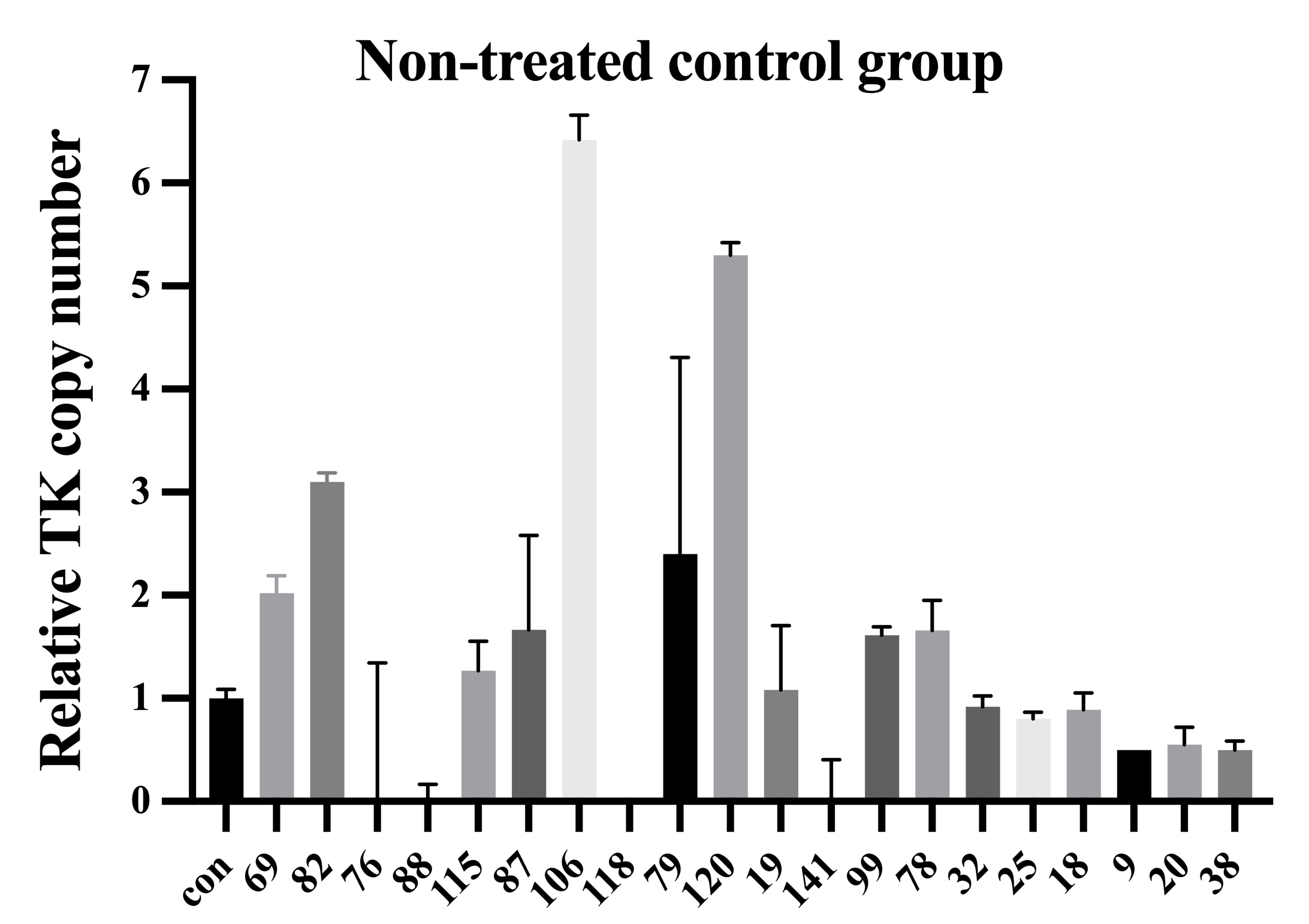


**Supplementary Figure 10.** **The relative copy number of thymidine kinase (TK) in non-treated control group.** The error bars represent the standard deviations (n = 2).


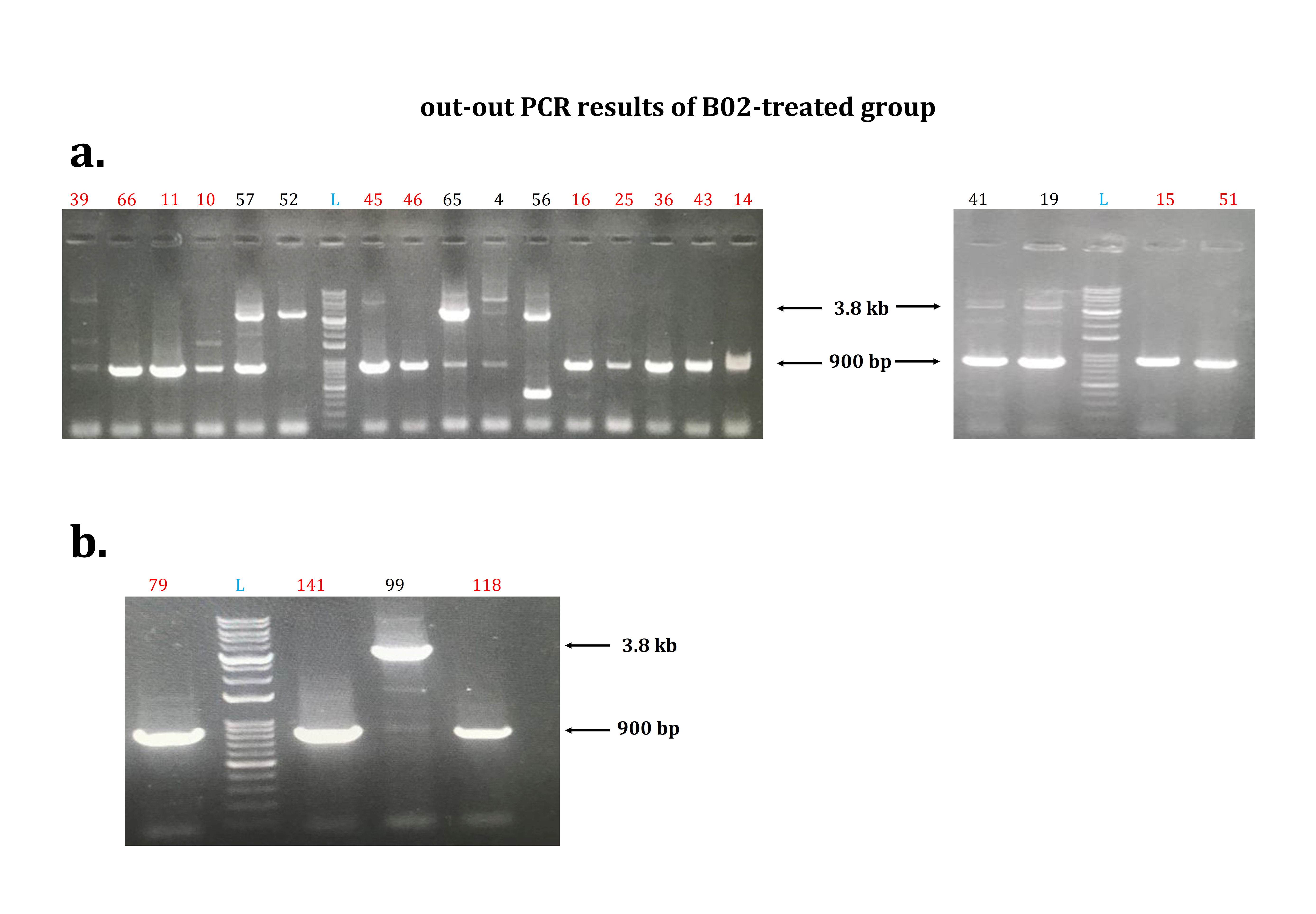


**Supplementary Figure 11. Agarose gel electrophoresis of out-out PCR results of single-cell clones of B02-treated (a) and non-treated control (b) groups.** The locus annealing primers were used in the PCR reaction to amplify the targeted cassette. The expected PCR product band of targeted locus is 3800. The out-out PCR negative clones were depicted in red (they just show wild-type CHO band (900 bp)).


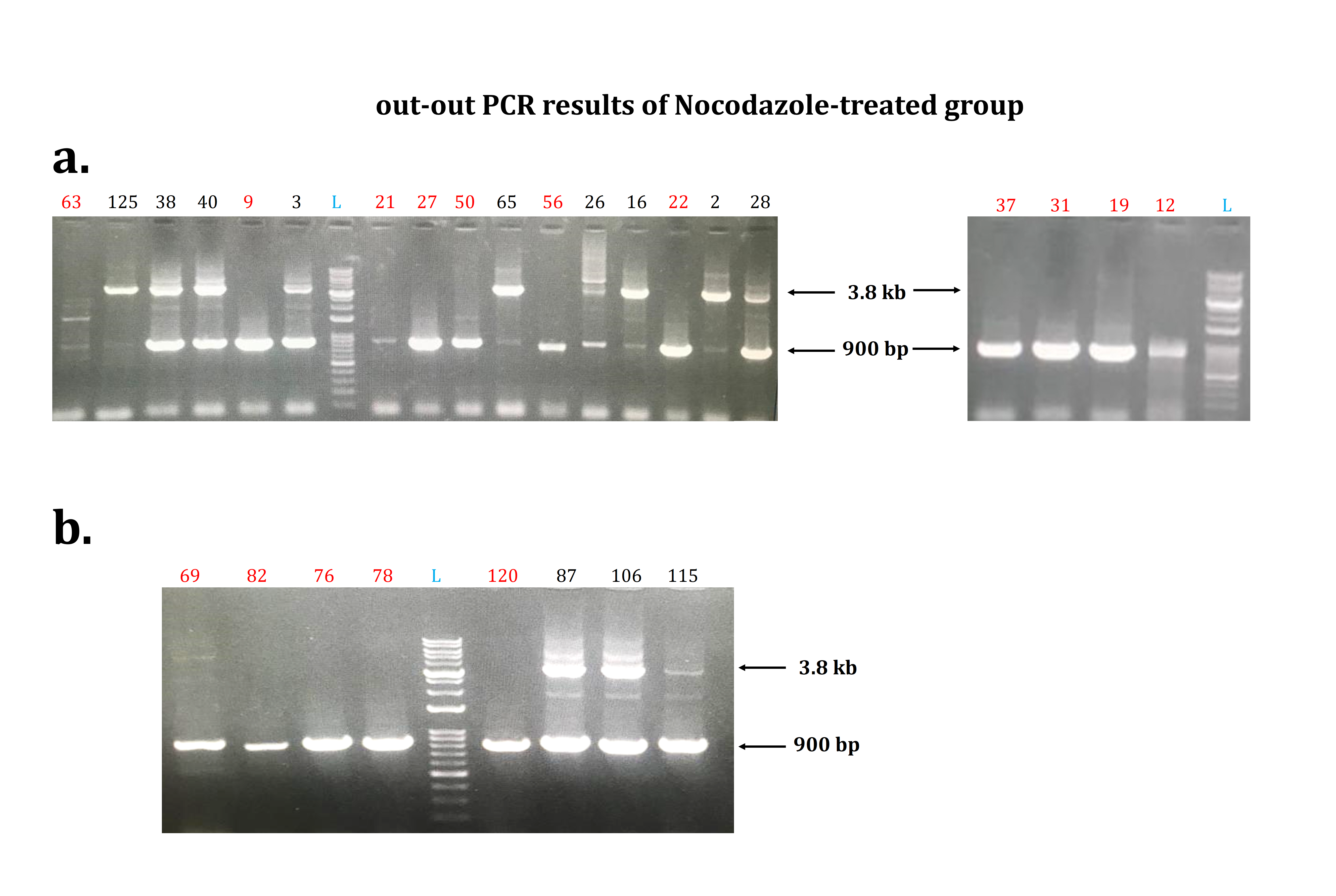


**Supplementary Figure 12. Agarose gel electrophoresis of out-out PCR results of single-cell clones of Nocodazole-treated (a) and non-treated control (b) groups.** The locus annealing primers were used in the PCR reaction to amplify the targeted cassette. The expected PCR product band of targeted locus is 3800. The out-out PCR negative clones were depicted in red (they just show wild-type CHO band (900 bp)).

**
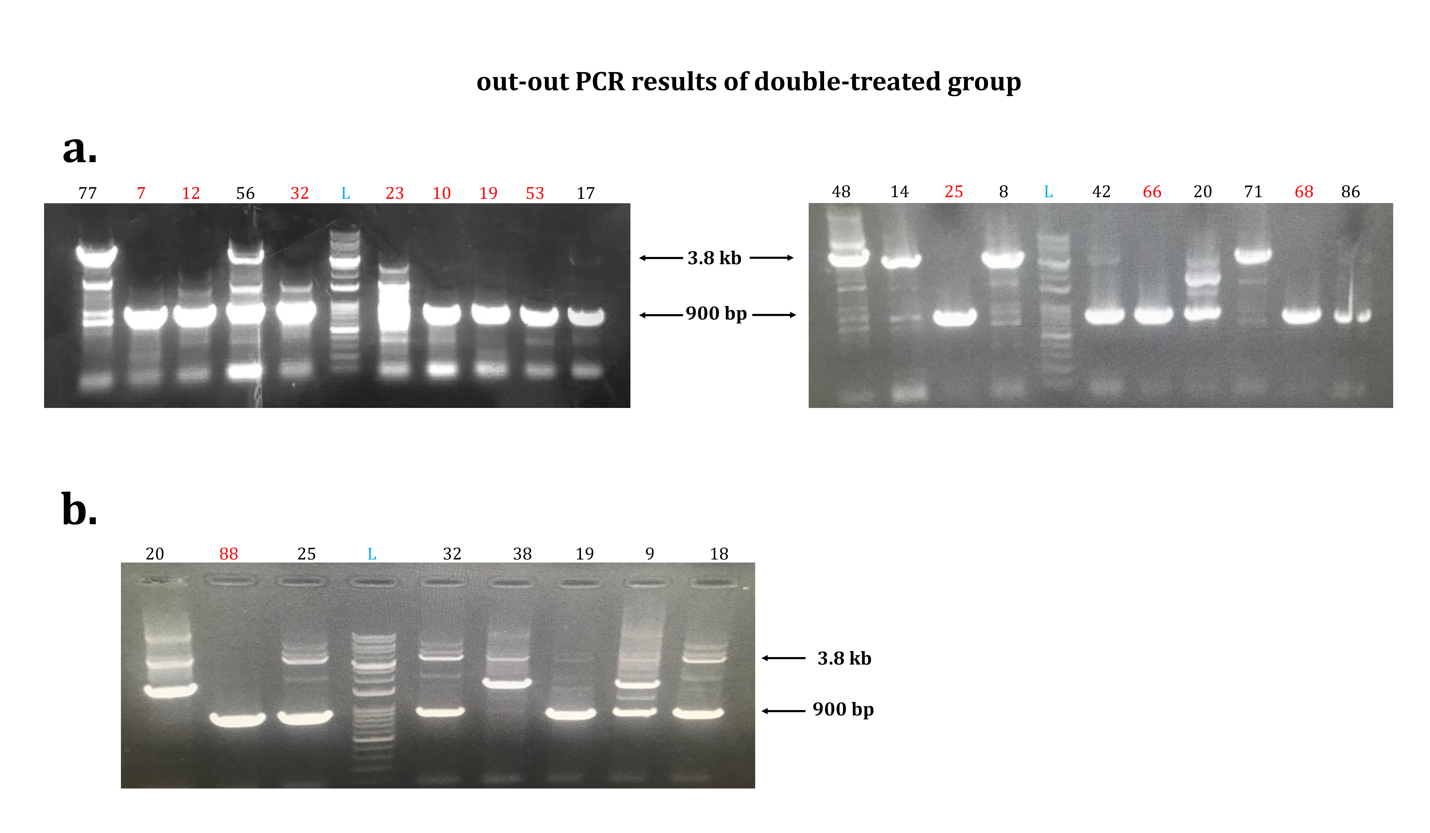
**

**Supplementary Figure 13. Agarose gel electrophoresis of out-out PCR results of single-cell clones of double-treated (a) and non-treated control (b) groups.** The locus annealing primers were used in the PCR reaction to amplify the targeted cassette. The expected PCR product band of targeted locus is 3800. The out-out PCR negative clones were depicted in red (they just show wild-type CHO band (900 bp)).


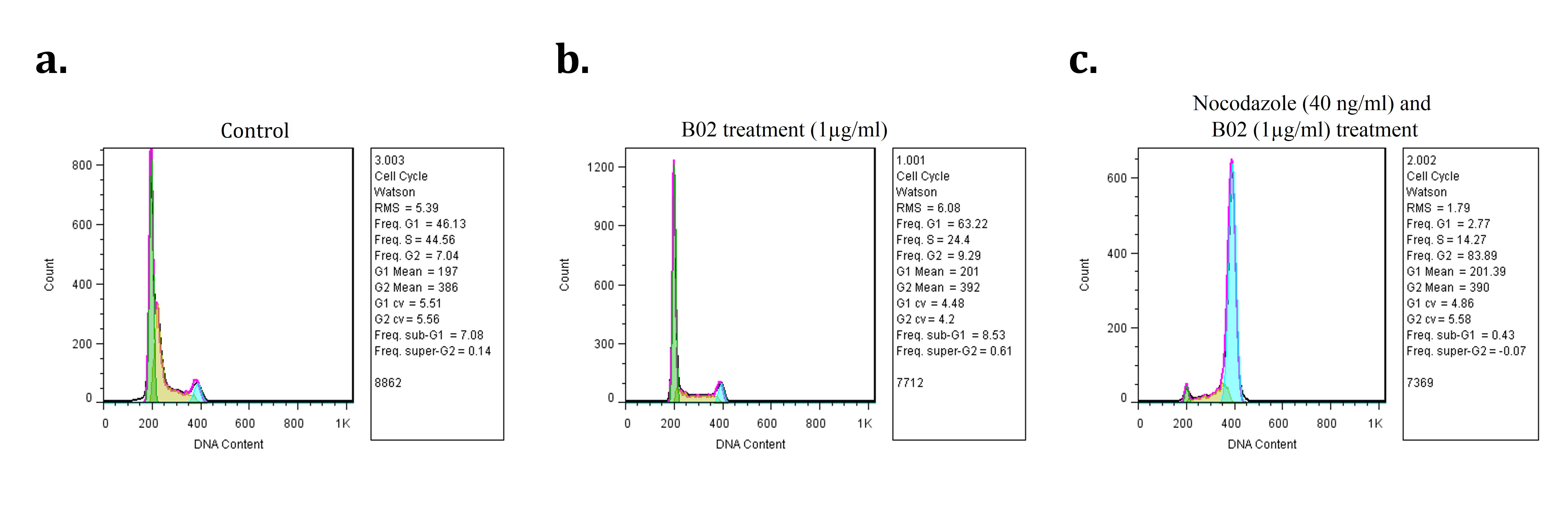


**Supplementary Figure 14. Cell cycle synchronization analysis following B02 and Nocodazole and B02 (double) treatments by Flow cytometry assay. (a)** The CHO-K1 cell without any treatment was used as a control. **(b)** Treatment of cells with 1µg/ml B02 for 48 hours following transfection. **(c)** Treatment of cells with 1µg/ml B02 for 48 hours and 40 ng/ml Nocodazole for 15 hours following transfection (Nocodazole was added to the cells 33 hours post-transfection, it means the cells were treated with both molecules for 15 hours).

**Supplementary Table 1. The sequence of locus-targeting sgRNAs**

| **No.** | **Target sequence**  **(5’ 3’)** | **PAM** |
| --- | --- | --- |
| **sgRNA1** | CCTTTCACGACTACTTCACTTGT | CCT |
| **sgRNA2** | CCACTCAGTAGCCTTTCACGACT | CCA |

**Supplementary Table 2. The sequence of 5' and 3' junction PCR primers**

| **No.** | **Primer name** | **Function** | **5' to 3' sequence** |
| --- | --- | --- | --- |
| **1** | 5' junction F | 5' junction amplification and out-out PCR | TTCGTTTAACAAAGCTAAAATGCCT |
| **2** | 5' junction R | 5' junction amplification and sequencing | CCCAGAAAGCGAAGGAGCAAA |
| **3** | 3' junction F | 3' junction amplification | GCAGCAACAGATGGAAGGCCT |
| **4** | 3' junction R | 3' junction amplification and sequencing and out-out PCR | GAAACCAAGGGCCACGGTAA |

**Supplementary Table 3. The sequence of out-out PCR primers**

| **No.** | **Primer name** | **Function** | **5' to 3' sequence** |
| --- | --- | --- | --- |
| **1** | 5' junction F | Out-out PCR | TTCGTTTAACAAAGCTAAAATGCCT |
| **2** | 3' junction R | Out-out PCR | GAAACCAAGGGCCACGGTAA |

**Supplementary Table 4. The sequence of primers used in real-time PCR**

| **No.** | **Primer name** | **Function** | **5' to 3' sequence** |
| --- | --- | --- | --- |
| **1** | TK F | TK amplification for qRT-PCR | AGCAGAAAATGCCCACGCTA |
| **2** | TK R | TK amplification for qRT-PCR | AGTAAGTCATCGGCTCGGGT |
| **3** | Beta-actin F | Beta-actin amplification for qRT-PCR | CTACGCTCTCCCTCATGCCATC |
| **4** | Beta-actin R | Beta-actin amplification for qRT-PCR | ATGTCACGCACAATTTCCCTCTC |


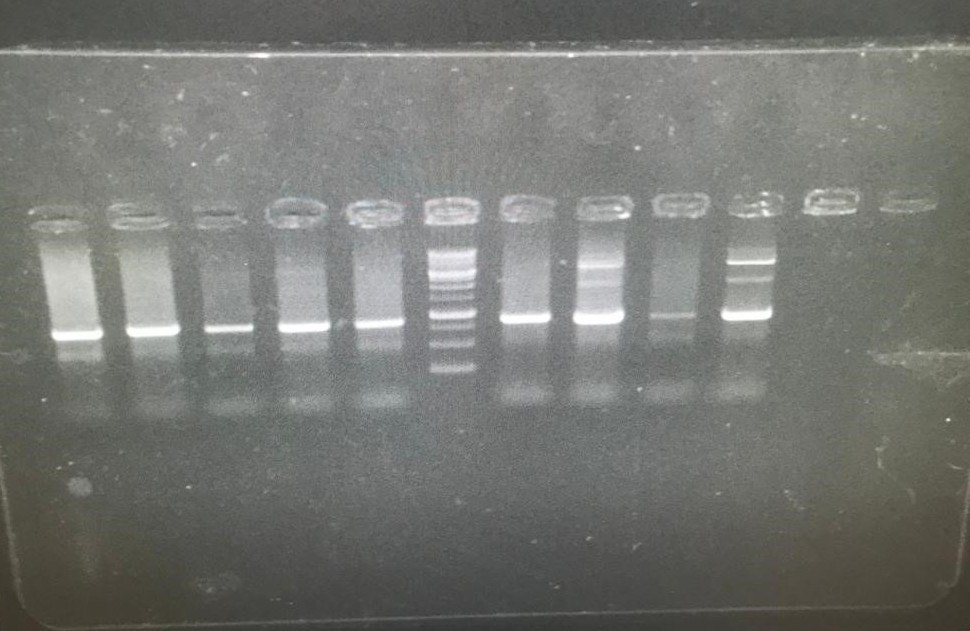


**Supplementary Figure 15a.** The original image of **figure 1d** (left-hand image; 5’ junction). The related lanes were depicted by red bracket


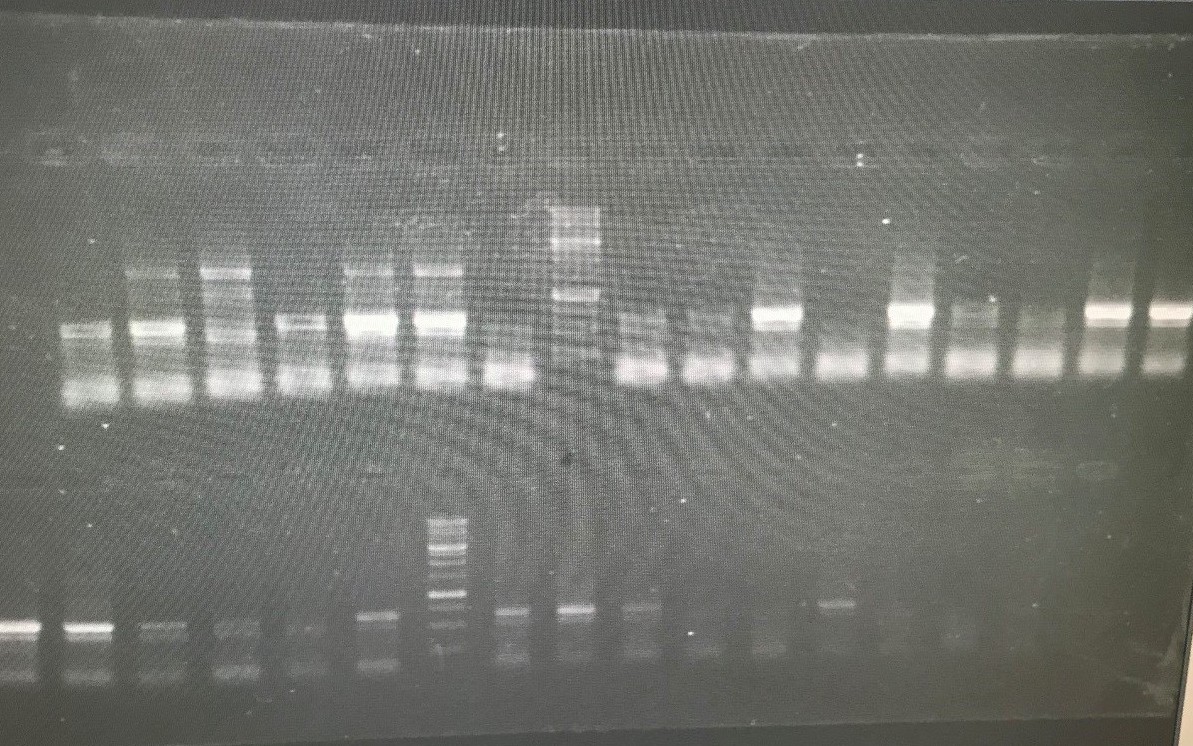


**Supplementary Figure 15b.** The original image of **figure 1d** (right-hand image; 3’ junction). The related lanes were depicted by red bracket


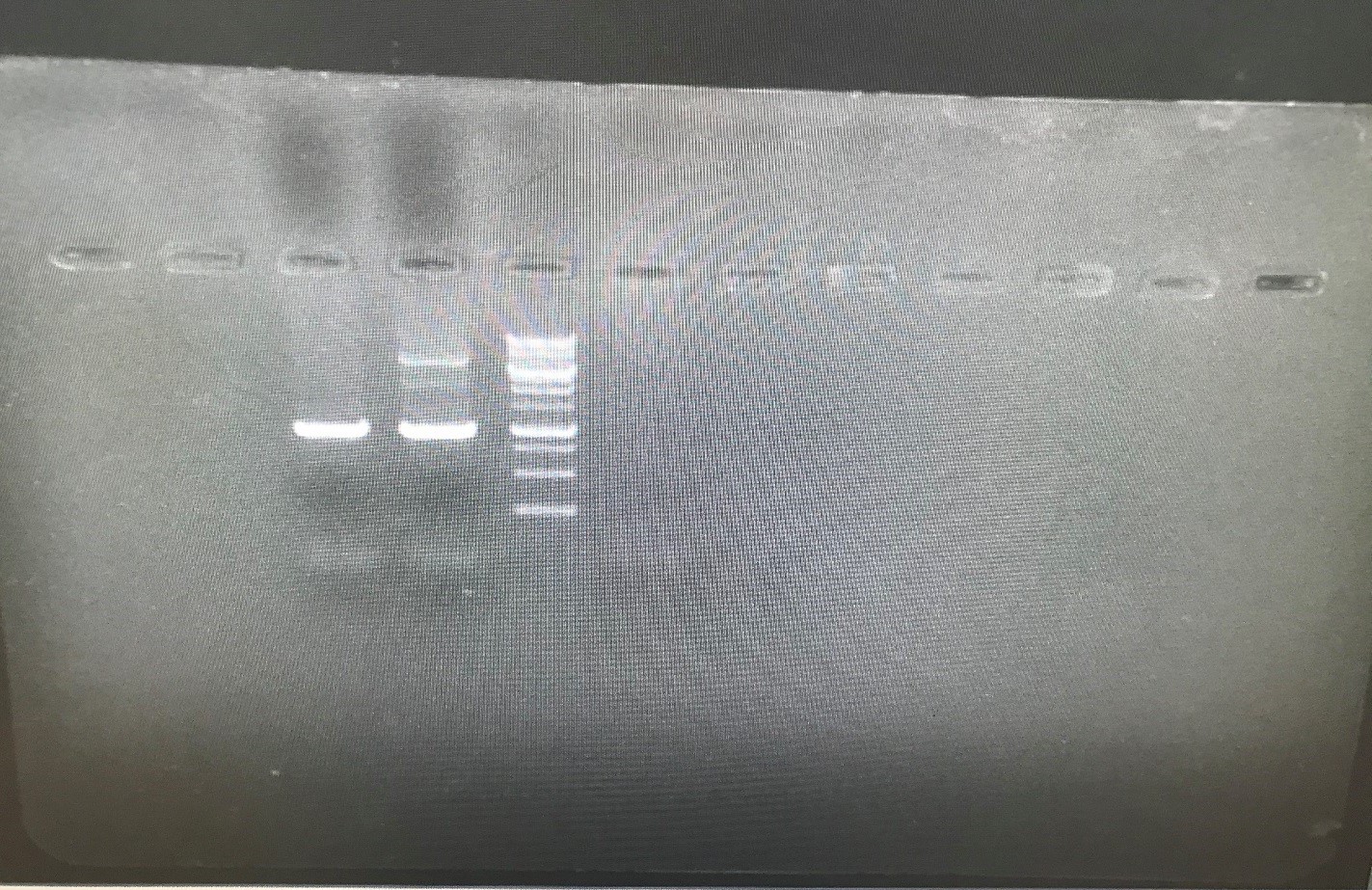


**Supplementary Figure 15c.** The original image of **figure 1e**. The related lanes were depicted by red bracket


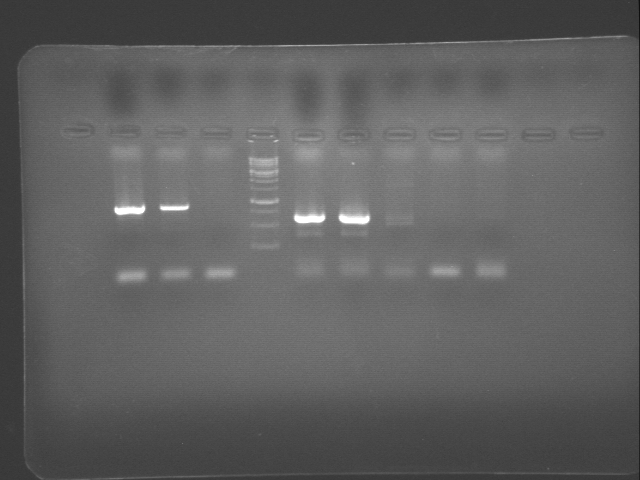


**Supplementary Figure 15d.** The original image of **figure 2b**. The related lanes were depicted by red bracket


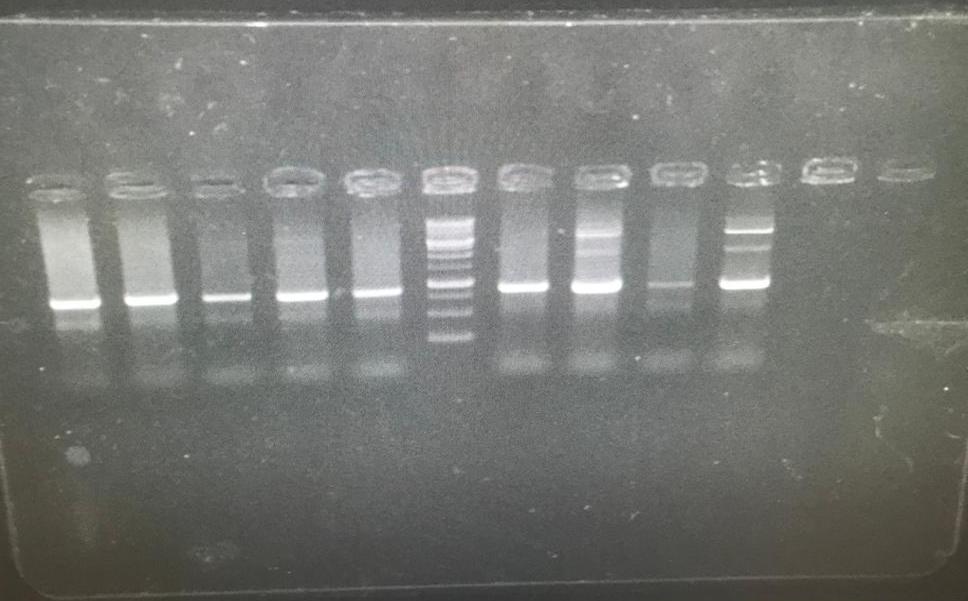


**Supplementary Figure 15e.** The original image of **figure 2c**. The related lanes were depicted by red brackets


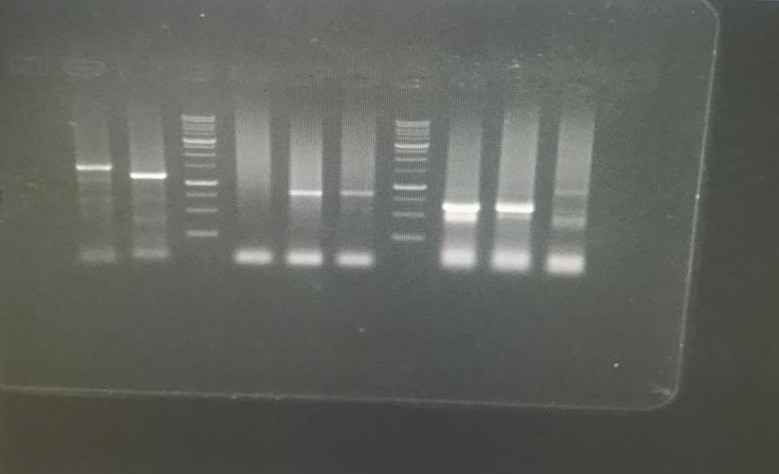


**Supplementary Figure 15f.** The original image of **figure 3d**. The related lanes were depicted by red bracket


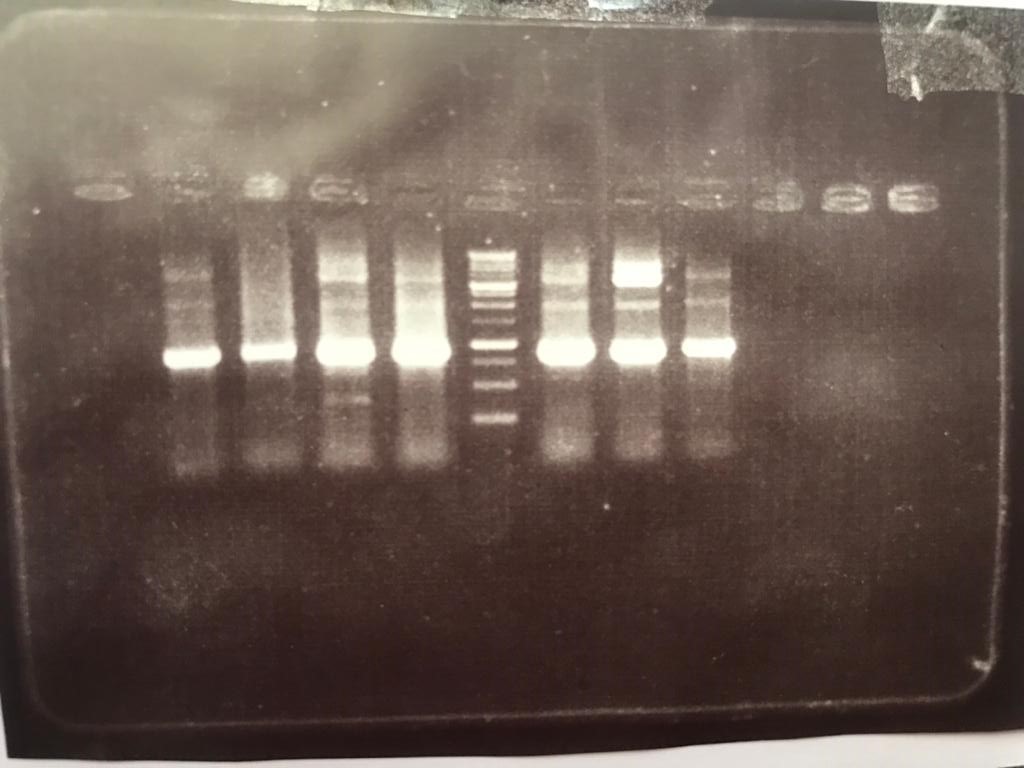


**Supplementary Figure 15g.** The original image of **figure 3e**. The related lanes were depicted by red bracket
